# Spontaneous cortical activity is transiently poised close to criticality

**DOI:** 10.1371/journal.pcbi.1005543

**Published:** 2017-05-24

**Authors:** Gerald Hahn, Adrian Ponce-Alvarez, Cyril Monier, Giacomo Benvenuti, Arvind Kumar, Frédéric Chavane, Gustavo Deco, Yves Frégnac

**Affiliations:** 1 Unité de Neuroscience, Information et Complexité (UNIC), CNRS, Gif-sur-Yvette, France; 2 Center for Brain and Cognition, Computational Neuroscience Group, Department of Information and Communication Technologies, Universitat Pompeu Fabra, Barcelona, Spain; 3 Institut des Neurosciences de la Timone, CNRS, Marseille, France; 4 Bernstein Center for Computational Neuroscience, Freiburg, Germany; 5 Dept. of Computational Science and Technology, School of Computer Science and Communication, KTH, Royal Institute of Technology, Stockholm, Sweden; 6 Institució Catalana de la Recerca i Estudis Avançats, Universitat Pompeu Fabra, Barcelona, Spain; 7 Department of Neuropsychology, Max Planck Institute for Human Cognitive and Brain Sciences, Leipzig, Germany; 8 School of Psychological Sciences, Monash University, Melbourne, Clayton, Victoria, Australia; Hamburg University, GERMANY

## Abstract

Brain activity displays a large repertoire of dynamics across the sleep-wake cycle and even during anesthesia. It was suggested that criticality could serve as a unifying principle underlying the diversity of dynamics. This view has been supported by the observation of spontaneous bursts of cortical activity with scale-invariant sizes and durations, known as neuronal avalanches, in recordings of mesoscopic cortical signals. However, the existence of neuronal avalanches in spiking activity has been equivocal with studies reporting both its presence and absence. Here, we show that signs of criticality in spiking activity can change between synchronized and desynchronized cortical states. We analyzed the spontaneous activity in the primary visual cortex of the anesthetized cat and the awake monkey, and found that neuronal avalanches and thermodynamic indicators of criticality strongly depend on collective synchrony among neurons, LFP fluctuations, and behavioral state. We found that synchronized states are associated to criticality, large dynamical repertoire and prolonged epochs of eye closure, while desynchronized states are associated to sub-criticality, reduced dynamical repertoire, and eyes open conditions. Our results show that criticality in cortical dynamics is not stationary, but fluctuates during anesthesia and between different vigilance states.

## Author summary

Cortical activity spontaneously displays a large diversity of dynamics in different behavioral states and at multiple spatiotemporal scales. In the last decades, a unifying principle has been proposed to underlie the diversity and scale-invariance of brain dynamics: Criticality, a particular state of complex systems in which order and disorder coexist. On the other hand, the cortex can exhibit a continuum of states with different levels of collective synchrony and LFP fluctuations. Here, we ask how criticality measures vary as the cortex spontaneously fluctuates across different states. Using recordings from the primary visual cortex of the anaesthetized cat and awake monkey, we show that spiking patterns of cortical states differ in their proximity to criticality and their relation to the vigilance state.

## Introduction

The cortex continuously generates coordinated ongoing patterns of activity during varying behavioral states such as restful wakefulness [1], focused attention [2], sleep and anesthesia [3]. Patterned spontaneous activity has been reported at multiple scales as revealed by multi-electrode arrays [4], optical imaging [5,6] magnetencephalography [7] and functional MRI [1]. In spite of early findings that the statistics of cortical activity are highly variable [8], interest arose to find an overarching physical principle that explains in a unified way the dynamical organization of the brain. Theoretical reasoning made criticality, a concept borrowed from statistical physics, a plausible candidate for such a unifying concept as it has been proposed to account for the brain’s inherent complexity necessary to process and represent its environment [9]. In the critical regime, cascading dynamics are expected to produce a great diversity of transient co-activations or “neuronal avalanches” [10,11]. Within this framework, power laws and high levels of long-range correlations are two strong indicators of critical dynamics [11,12].

Even though first reported in *in vitro* preparations [13–18], evidence for criticality in the cortex was found during anesthesia [19,20], sleep [21], resting state in animals and humans [22–27], task-related conditions [28] and stimulus evoked activity [29,30]. However, other studies failed to find the typical signs of criticality, either refuting the neuronal avalanche hypothesis [31–33] or explaining the negative result by subsampling of neuronal activity due to fundamental measurement limitations of current multi-electrode recording techniques [21,28,34]. Alternatively, criticality or its absence in the cortex may not be a constant feature of brain activity, but fluctuates with the ever-varying dynamics of the cortex and associated arousal states. Indeed, the statistics of neuronal population spike activity in the cortex substantially vary over time, continuously moving between highly synchronized burst dynamics and desynchronized, irregular activity [35]. Synchronized cortical states are the hallmark of *in vitro* preparations [13], but also appear during slow wave sleep [36], anesthesia [21,37,38] and also in awake animals during quiet waking and drowsiness [39,40]. In contrast, desynchronized activity is associated with active behavior [41,42], responses to visual stimuli [43] and suggested to be a neuronal correlate of attention [35]. In line with this hypothesis, putative transitions between critical, supercritical and subcritical dynamics have been observed during anesthesia [20,44], shifts between anesthesia and the physiological sleep-wake cycle [21,22,45–48], prolonged waking [49], and in a rest versus task setting [50]. Here, we tested whether the signature of criticality at the level of collective spiking activity remains robust across different cortical states or fluctuates together with changing cortical dynamics recorded in the primary visual cortex of the anesthetized cat and awake monkey using classical criticality markers and data modeling. We found time varying criticality properties which were associated with the local cortical state, both during anesthesia and in the awake state, and changed with prolonged epochs of eye closure in the awake condition.

## Results

**Fig 1:**
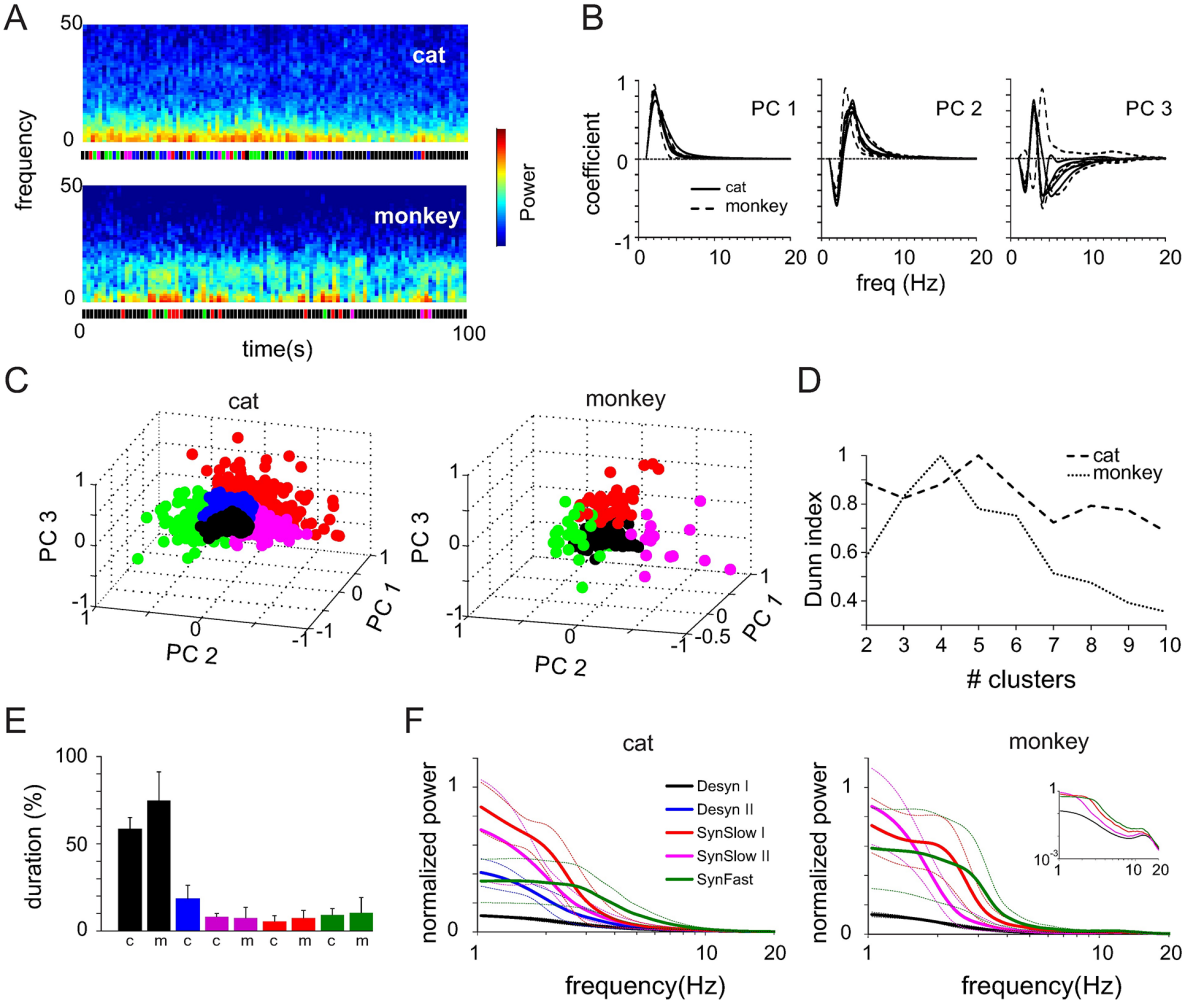
Separation of cortical states in spontaneous activity of anesthetized cat and awake monkey. **(A)** LFP spectrograms of two 100s segments computed with non-overlapping windows of 1s. Bottom: colored bars indicate cortical state as defined in the main text. **(B)** Coefficients for first three principal components as a function of power spectrum frequency. **(C)** Principal component space for two entire datasets (cat: 6000s, monkey: 600s). Each circle represents a data segment of 1s duration. Colors indicate different cortical states. (**D**) Dunn index as a function of the cluster number extracted by k-means. **(E)** Average (+SD) duration of different states across all datasets of a species. **(F)** Average power spectrum of different cortical states for all cat and monkey datasets. Dashed lines indicate standard deviation (±SD). Inset: same as in main figure, but in log-log coordinates to show peak in alpha band.

In this study, we recorded spontaneous spiking activity and local field potentials (LFP) from the primary visual cortex of four anesthetized cats (~1 hr/animal) and one awake monkey using 32-channel silicon probes (cats) and a chronically implanted 96-channel Utah array (monkey, 4x10 minutes, total of 40 minutes). To avoid interference of visually evoked activity with the ongoing dynamics all recordings were performed in the dark. Visual inspection of the LFP spectrogram of both cat and monkey datasets showed non-stationary dynamics with prominent power fluctuations in a frequency band between 1 and 15 Hz and stronger alpha oscillations (9–13 Hz) in the monkey recordings (Fig 1A).

### State separation

In order to account for the variability of cortical dynamics, we attempted to pool short segments of the data into sets which were characterized by a similar frequency composition of the LFP power spectrum. Each set is referred to as a different cortical (or dynamical) state in accordance with previous studies [35,38,41].

We first used a combination of principal component analysis (PCA) and subsequent k-means clustering to find frequency bands that showed the highest variance across an entire dataset and subsequently cluster data epochs with similar power in these bands (see Materials and methods). The first three principal components (PC) explained 97% (cats) and 99% (monkey) of the variance and corresponded to different bands of the frequency spectrum within a range between 1 and 15 Hz (Fig 1B). Each data segment was represented in a three dimensional PC space and we observed a characteristic pattern of segment clusters across cat and monkey datasets. Most segments were concentrated at the origin of the PC space around which the remaining ones were scattered along the three dimensions (Fig 1C). We performed a k-means cluster analysis to extract different clusters in each dataset. The optimal number of clusters was estimated using a validation procedure based on the Dunn index (DI). On average, the optimal number of clusters, as indicated by a peak in the DI distribution, was found to be five in the cat datasets and four in the monkey recordings (Fig 1D), each of which corresponded to different cortical states. In order to compare states across different datasets and species, we coded clusters at the same location in the PC space with the same color (see color code in Fig 1C). Analysis of cluster duration revealed that the cortex spent most of the time in the cluster centered around the origin of the PC space (58 ± 0.07% and 75 ± 0.17%, mean ± SD of the data in the cat and monkey, respectively), while cortical dynamics represented in the other clusters was much less frequent (Fig 1E).

Next, we computed average power spectra across all data segments in one cluster and the analysis results confirmed that the separation algorithm extracted power spectra with a similar frequency composition across different time epochs (Fig 1F). The black clusters consistently corresponded to dynamics with little power in lower frequencies (1–5 Hz) as opposed to the red and green clusters in which the power in slower (red) or faster frequencies (5–15 Hz, green) was high. The blue and magenta clusters had intermediate power at slower frequencies approaching either the black or red cluster, respectively. In the subsequent analysis we will refer to states with relatively lower power in slow frequencies as “desynchronized states”—denoted as Desyn I (black) and Desyn II (blue, only for cat)—, with higher power in slow frequencies as “slow synchronized states”—denoted as SynSlow I (magenta) and SynSlow II (red)—and with high power in faster frequencies as “fast synchronized state”—denoted as SynFast (green).

### Characteristics of spiking activity across cortical states

**Fig 2:**
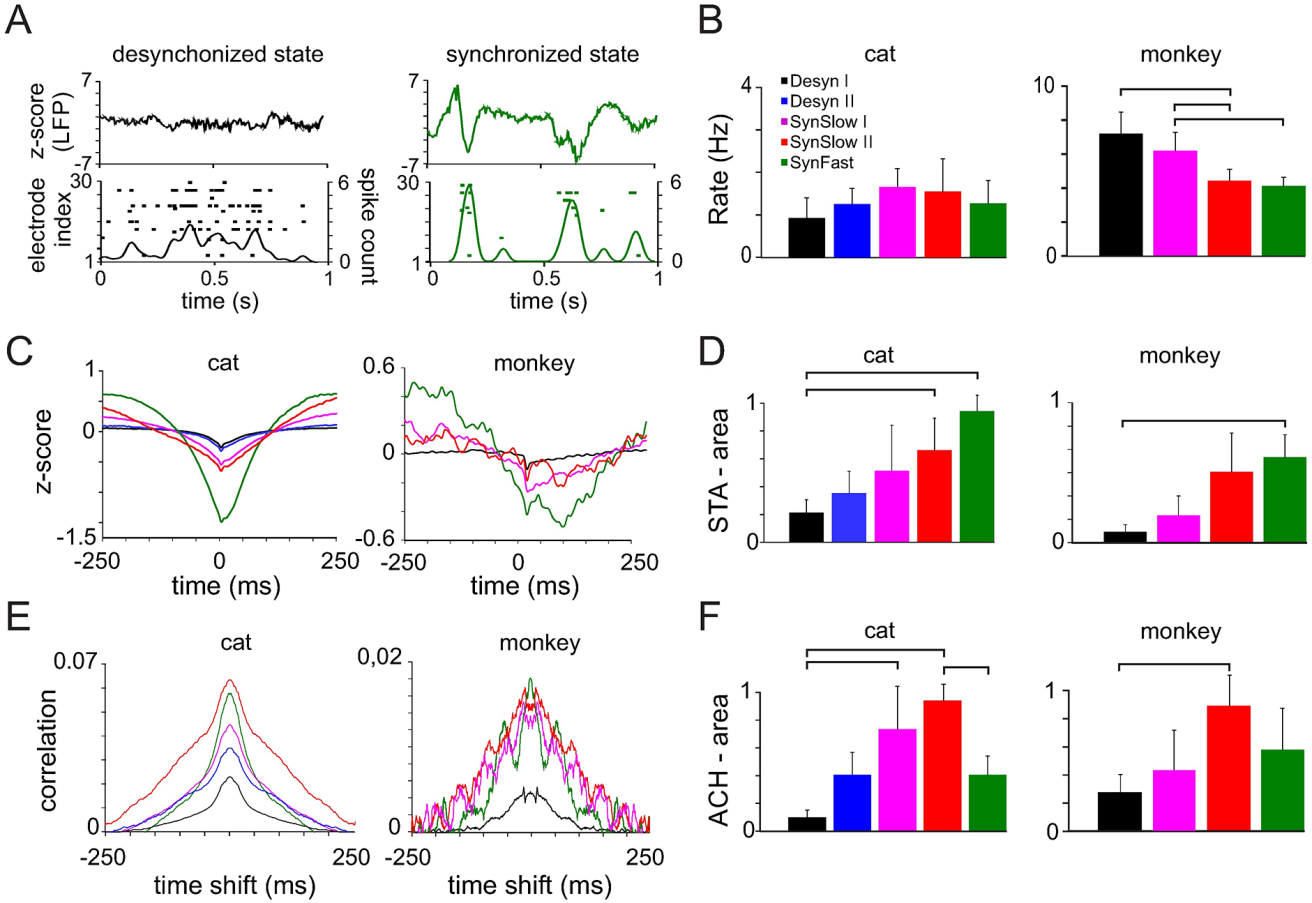
Characteristics of LFP-spike relationship and spiking activity for different cortical states. **(A)** LFPs (top) and spike rasters (bottom) for 1s segments of a desynchronized and synchronized cortical state from one cat dataset. Spike counts were computed with a Gaussian kernel (20ms window-size). **(B)** Average (+SD) population firing rate for all recordings of a species. **(C)** Examples of spike-triggered averages (STA) for different cortical states. (**D**) Average (+SD) STA area across all cat and monkey datasets. The area is normalized to the state with the largest value within a dataset. **(E)** Autocorrelation histograms of the population spike trains (pACH) for all cortical states in one cat and one monkey recording. (**F**) Average (+SD) area and of ACH peaks computed for all cat and monkey datasets. The values were normalized to the states with maximum area within a dataset. Horizontal bars: significant Bonferroni multiple comparison test (p<0.05).

Spiking activity consistently varied with the cortical states as defined by the LFP (see Fig 2A for an example taken from a cat recording). During epochs of desynchronized LFP with small amplitude fluctuations, spiking activity was characterized by continuous and irregular firing of neurons without apparent synchronization across the different channels. When the LFP shifted to dynamics with larger amplitude fluctuations, the underlying firing pattern of neurons was characterized by bursts of spiking activity that occurred synchronously across most of the channels and was followed by periods of silence.

The mean channel firing rates consistently varied across different states (Fig 2B). Neuronal activity in the anesthetized cat showed a trend to increased firing rates as LFP entered into synchronized states, but differences across states were not significant (one-way rm-ANOVA: p > 0.11). In contrast, neurons in the awake monkey fired significantly more vigorously in desynchronized states than during synchronized activity (one-way rm-ANOVA: F_3,9_ = 39.03, p = 0.0005, *ε* = 0.62).

To further quantify the relationship between spikes and LFP we computed spike-triggered averages (STA) for each dataset and cortical state (see Fig 2C for two examples of a cat and a monkey dataset). In all cases, as expected, spikes were associated with the negative trough of LFP deflections, whose area between the negative deflection and the zero baseline (one-way rm-ANOVA: F_4,12_ = 7.26, p = 0.019, *ε* = 0.56 (cat) and F_3,9_ = 6.63, p = 0.027, *ε* = 0.71 (monkey)) varied between states. In desynchronized states the LFP peaks were small and thin, while they increased in amplitude and width in more synchronized states. The synchronized cluster with faster deflections displayed peaks with the highest amplitude, but its width was smaller than synchronized states with lower frequency fluctuations. These results were confirmed across all datasets and both species (Fig 2D).

To assess the level of correlation in the population spiking activity, we computed the population auto-correlation histograms (pACH) of the population spike trains for each state (Fig 2E) which captures all possible correlations either generated intrinsically by neurons (i.e. bursting behavior) or through neuronal interactions. The area of pACH peaks was significantly state dependent (one-way rm-ANOVA: F_4,12_ = 15.91, p = 0.002, *ε* = 0.6 (cat) and F_3,12_ = 4.27, p = 0.018, *ε* = 1 (monkey)). The pACH of the Desyn I state had lower amplitude and area compared to the other states, while the pACHs of SynSlow II and SynFast states had the largest peaks. The pACHs of the slow synchronized states had the largest spread, while the temporal correlations were shortened in the other states. In addition, spike synchronization in faster frequency bands (alpha range) was clearly visible in the pACH of the monkey, especially in the SynFast state. These results were consistent across all datasets and species (Fig 2F).

### Cortical states and criticality

**Fig 3:**
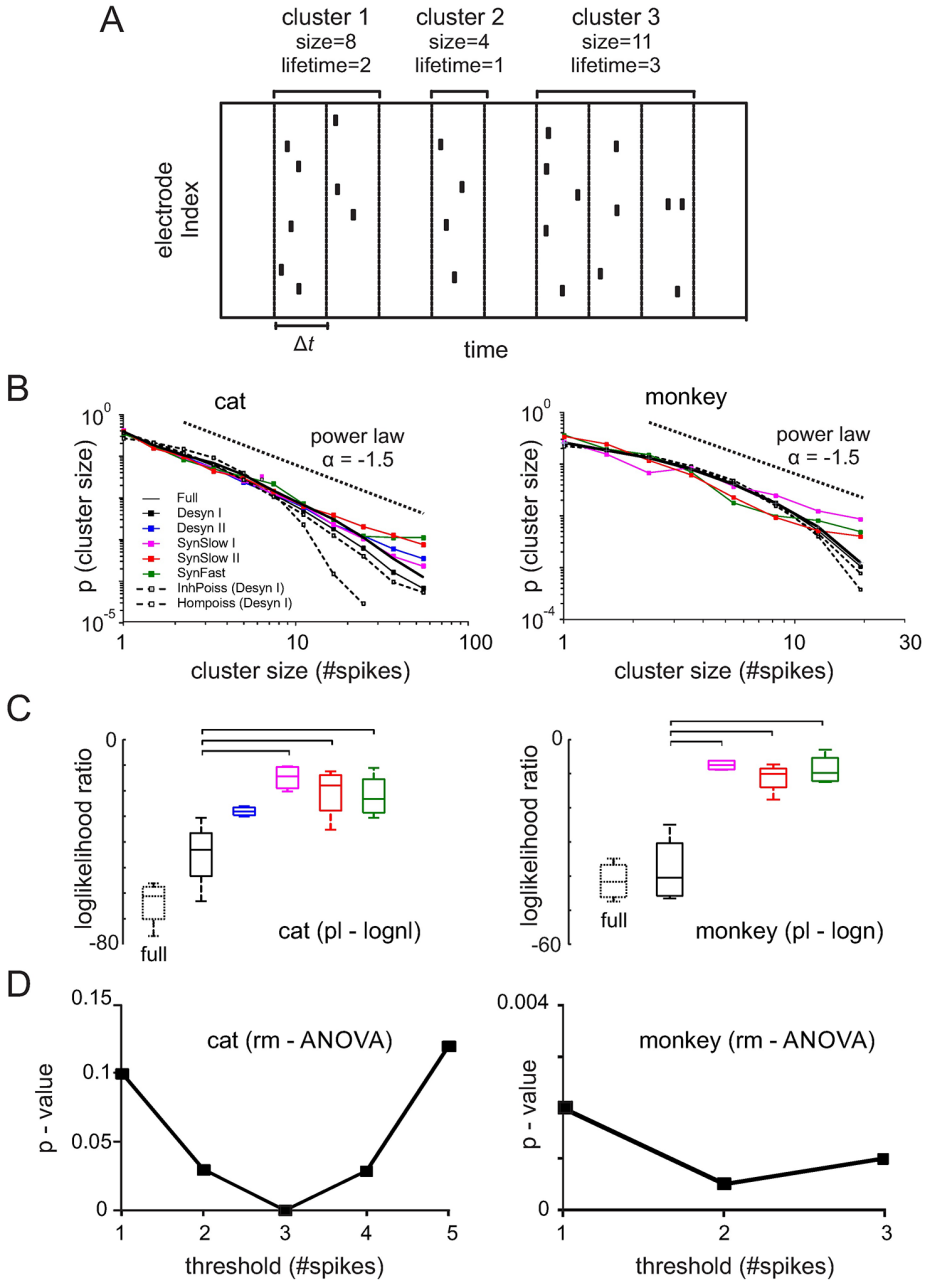
Neuronal avalanche analysis of spiking activity. **(A)** Spike clusters were defined as a sequence of bins containing spikes ≥1 threshold (in this example threshold = 1). The size of a spike cluster is given by the total number of spikes within a cluster. The lifetime of a cluster is defined as the number of bins. Δt was chosen as the average ISI_pop_ interval of the population spike train for each state. **(B)** Cluster size distributions of different states. Dotted black lines indicate power law with exponent = -1.5. Dashed lines with gray squares represent an inhomogenous Poisson process created from the desynchronized I state by spike time randomization within all 1s segments. Dashed lines with empty squares indicate homogenous Poisson process with the same rate and duration as the entire desynchronized I state. **(C)** Loglikelihood ratios for power law and lognormal fits to cluster size distributions of different cortical states across all cat (threshold: 3 spikes) and monkey data (threshold: 2 spikes). Negative values indicate a better lognormal fit. Horizontal bars: significant Bonferroni multiple comparison test. **(D)** Significance between different states expressed as the p-value of an rm-ANOVA test for different thresholds defining a cluster.

To study criticality in spiking activity, we first applied a classical analysis [13] that is based on binning point processes like spike trains and extracting clusters defined as consecutive bins with a number of spikes above a predefined threshold (Fig 3A, threshold ≥ 1). Bin-sizes were given by the average inter-spike interval of each state (see Table 1 for detailed values). Sufficiently sampled critical dynamics underlying the generated spikes predicts power laws in the cluster size distributions, i.e. a straight line in log-log plots. Such analysis was consequently performed for each cortical state separately and the full datasets for comparison (Fig 3B). The distributions in the most desynchronized states (black solid lines) did not follow a power law, albeit not being as curved as expected from shuffled data (Fig 3B, dashed lines). However, moving from desynchronized to more synchronized states, the tail of the cluster size distribution increased and came closest to a power law in the most synchronized states (red lines). This pattern was consistent in both cat and monkey recordings. Analysis of the full datasets revealed clearly curved distributions similar to the desynchronized state in accordance with previous studies in spiking data [21,31,32,51](Fig 3B, solid black line)

**Table 1:** Average inter-spike intervals (in ms) of the population spike train for all cat and monkey datasets.

Cortical State:	Cat				Monkey			
	Dataset 1	Dataset 2	Dataset 3	Dataset 4	Dataset 1	Dataset 2	Dataset 3	Dataset 4
**Desyn I**	14	43	28	30	27	14	21	16
**Desyn II**	26	31	23	43	--	--	--	--
**SynFast**	16	47	26	55	21	24	19	29
**SynSlow I**	56	38	27	22	25	22	30	19
**SynSlow II**	16	54	18	37	24	15	31	28
**Full**	28	48	26	45	23	15	21	16

To quantify these tails, we fitted both lognormal and power law distributions to the cluster size distributions. Lognormal functions are more flexible and support different degrees of heavytaildness as opposed to power laws with extreme tails. Thus, comparing the goodness of fit between both distributions provides a relative quantification of how closely a distribution approaches a power law. We performed comparisons using loglikelihood ratios (LLR) with positive values indicating superior power law fit and negative values giving more evidence for lognormal fits with reduced tails [22,52]. In our recordings, LLRs were in general negative across all states in both cat and monkey datasets (all datasets: p < 0.001), indicating that tails were less pronounced than expected by a power law. However, how much each state deviated from a power law varied between states as indicated by the absolute values of the LLR. These were more negative during desynchronized states and when analyzing the full datasets, while the discrepancy between lognormal and power law fit was less pronounced during states with more synchronization (Fig 3C). We repeated this analysis using different thresholds for avalanche detection (see Methods and material)[22,53]. In the cat, significant state differences (rm-ANOVA: p < 0.05) were found for all but the lowest and highest thresholds, which only yielded a trend (p < 0.1) or were non-significant, respectively. In the monkey, state differences were found for all thresholds (see example in Fig 3D and S1 Fig for a detailed analysis). We also determined the exponent of the fitted power law distributions for all states which yielded values for each species close to the theoretically expected and experimentally found value [13,54] of -1.5 (cats: -1.62 ± 0.05, monkey: -1.58 ± 0.03), desynchronized states included.

Even though power laws can be predictive of criticality, a failure to detect straight lines does not entirely rule out absence of criticality [21,28]. Another test for criticality is the existence of universal scaling functions that capture the system dynamics at different scales. In cortical cultures, it has been observed that the time-courses of long avalanches are scaled versions of short avalanches as predicted close to a critical point [15]. Such invariance across scales, or self-similarity, known as “shape collapse”, was also found in spiking data of rats undergoing all stages of the sleep-wake cycle and resting state EEG recordings of humans [21,50]. Here, we used the method of Marshall et al. [55] to examine whether avalanches time-courses are self-similar for the different states and show shape collapse (for details see Supplementary Material). Since this analysis requires a large amount of data, we restricted our analysis to the cat data. The goodness of collapse was quantified by the collapse index (CI), defined as the variance across re-scaled avalanche profiles. We found that the empirical avalanche profiles collapse significantly (p = 0.027) more for the synchronized states (SynSlow I/II) than for the desynchronized states (Desyn I/II) (S3A–S3F Fig). These results are consistent with the power law analysis showing that the presence of curved avalanche size distributions is accompanied by a reduction in self-similarity and indicate stronger deviations from a critical state in desynchronized cortical dynamics.

### Predicting criticality from LFP fluctuations and spike correlations

**Fig 4:**
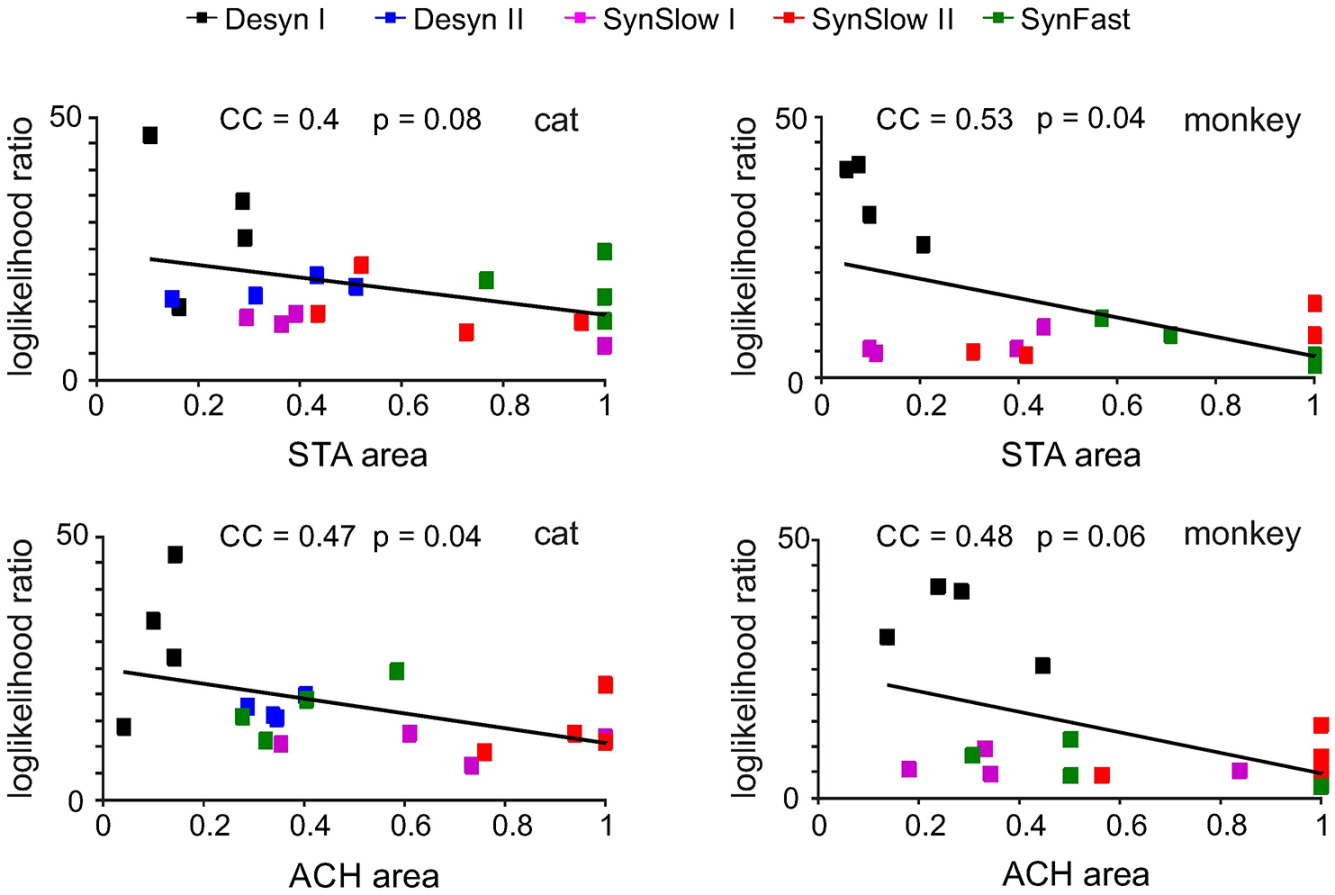
Correlation between LFP, spike synchronization and the level of criticality measured by the loglikelihood ratio for power law and lognormal fits. Top: LLR as a function of STA area for all cat and monkey datasets. Bottom: LLR as a function of ACH area.

Next, we attempted to synthesize the above findings by showing how different measures of LFP and spiking activity are linked and relate to criticality in neuronal discharges. To this end, we quantified the correlation between the loglikelihood ratio fitted to all spike cluster size distributions, and the mean autocorrelation between neurons (i.e. area of the population ACH, representing the total amount of correlation in the neuronal population) as well as the amount of spike-triggered LFP fluctuations (i.e. the average STA area across channels). As the STA and ACH peaks became larger, the tails of the cluster size distributions and the associated loglikelihood ratios significantly increased or followed a trend (Fig 4). This linear dependence indicates that the size of the STA and the level of population autocorrelation in spiking activity are directly translated into the degree of tailedness of the spike cluster size distributions. As the LFP varied with cortical state, the results of the neuronal avalanche analysis changed from more power law like to more curved distributions indicating the absence of correlated population activity.

### Lifetime distributions

**Fig 5:**
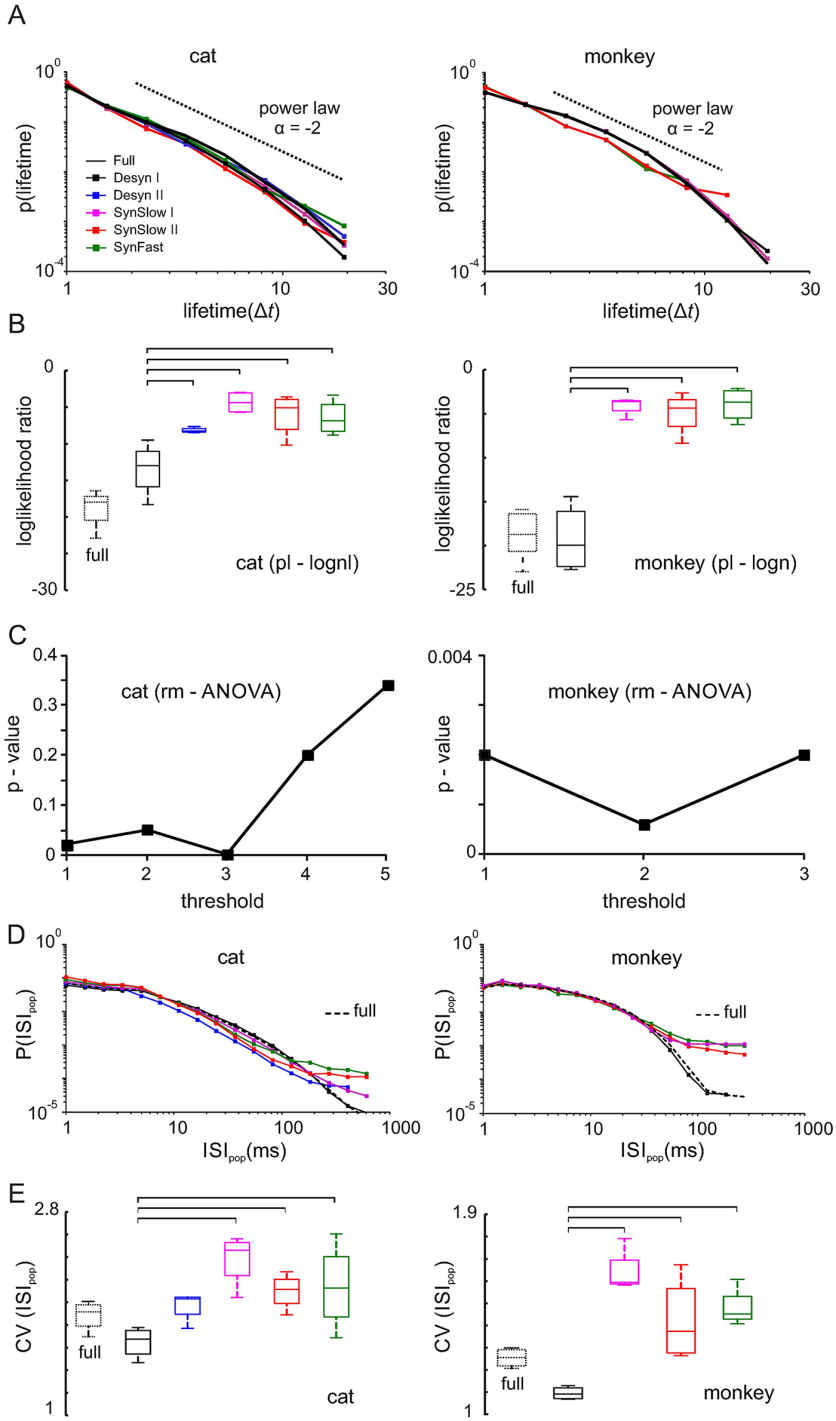
Lifetime and ISI_pop_ distributions of spiking activity. **(A)** Lifetime distributions of different states. Dotted black lines indicate power law with exponent = -2. **(B)** Loglikelihood ratios for power law and lognormal fits to lifetime distributions of different cortical states across all cat (threshold: 3 spikes) and monkey data (threshold: 2 spikes). Negative values indicate a better lognormal fit. Horizontal bars: significant Bonferroni multiple comparison test. **(C)** Significance between different states calculated as the p-value of an rm-ANOVA test for varying thresholds constituting a cluster. **(D)** ISI_pop_ distributions of population spike trains for one cat and one monkey recording. **(E)** Coefficient of variation for ISI_pop_ distributions of different states across all cat and monkey datasets.

Another signature of criticality is a power law in the distribution of lifetimes, i.e. the duration of neuronal event clusters (Fig 5A), which decays with a characteristic exponent of -2. This theoretically obtained result [54] was indeed found in neuronal networks in vitro and in vivo using LFP events and spiking activity [13,22]. Here, we also calculated lifetime distributions and assessed their closeness to power laws by comparing LLRs between power laws and lognormals across different states. Similar to the cluster size distributions, LLRs indicated a generally better lognormal fit (all datasets: p < 0.001). However, LLR values during synchronized states were significantly less negative during synchronized states compared to desynchronized states and the full datasets (Fig 5B). These differences were significant (rm-ANOVA: p<0.05) for thresholds one and three in the cat, and for all thresholds in the monkey (Fig 5C and S2 Fig). Exponents of fit power law distributions remained unchanged across different states and approached a value of -2 (cats: -1.84 ± 0.06, monkey: -1.74 ± 0.04), as predicted by theory and other experiments [13,54].

### Inter-spike interval distributions

Previous studies reported heavy tails in the inter-spike interval distributions of individual neurons [56,57] and spike trains of neuronal populations [20,58]. In this study, we also compared inter-spike interval distributions of population spike trains (ISI_pop_) for each state quantifying differences through the coefficient of variation (CV = std(ISI_pop_) / mean(ISI_pop_)). In both cat and monkey recordings the distributions of desynchronized states and the entire datasets were clearly curved (Fig 5D) and approached a CV of ~1 (Fig 5E), which is expected for independent Poisson processes. In contrast, synchronized states were associated with heavier tails as expressed by significantly larger CVs (one-way rm-ANOVA: F_4,12_ = 9.66, p = 0.03, *ε* = 0.35 (cats) and F_4,12_ = 18.18, p < 0.001, *ε* = 1 (monkey)).

### Temporal evolution of cortical states

**Fig 6:**
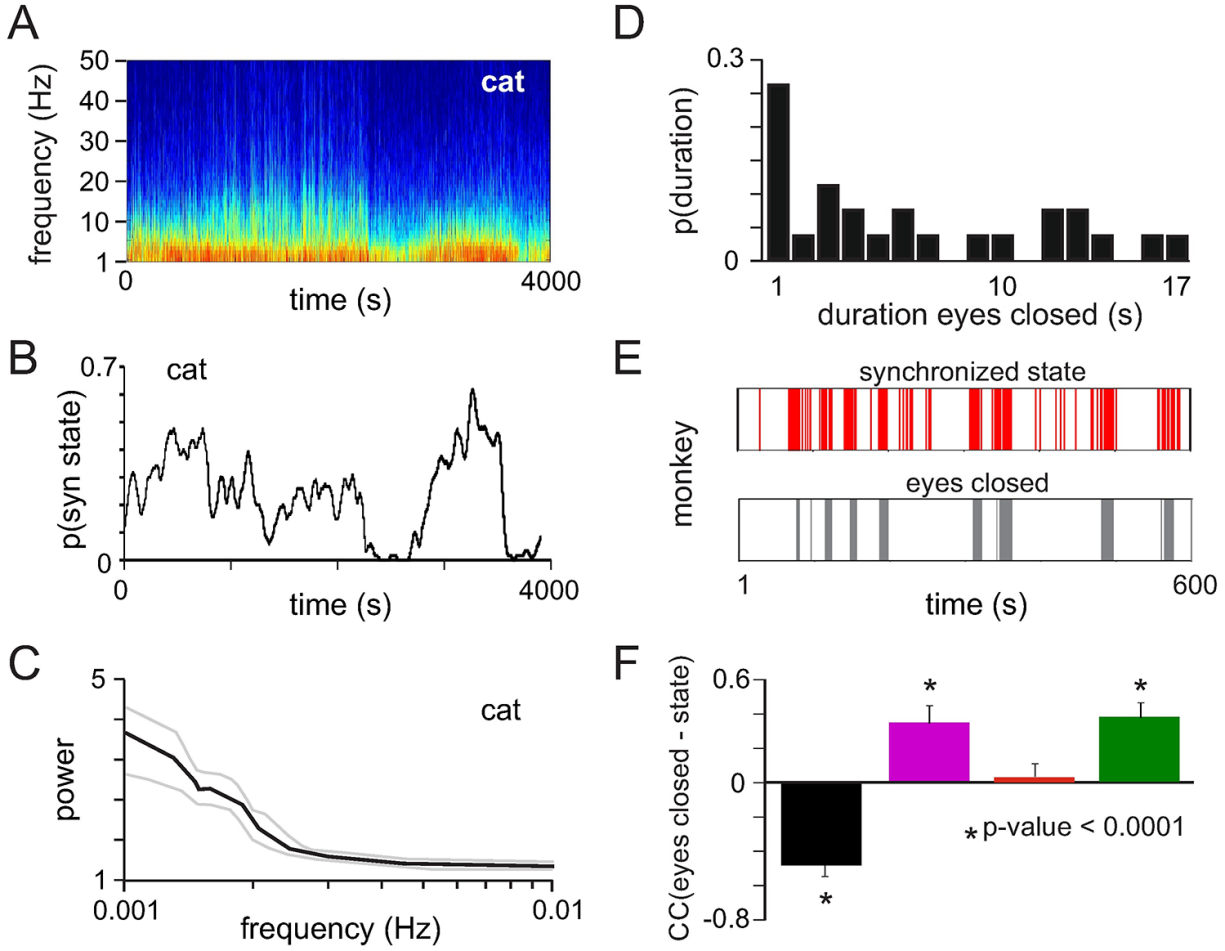
Evolution of cortical states over time. **(A)** LFP spectrogram of an entire recording in one cat calculated with non overlapping windows of 1s duration (top) and the probability of synchronized states (synchronized slow I, II and synchronized fast states) computed with a sliding window of size 100s and an overlap of 1s (bottom). **(B)** Average power spectrum (SEM: dashed lines) of the time courses of synchronized states across all four cats. **(C)** Probability to find periods of eye closure with a given duration across all monkey datasets. **(D)** Time course of synchronized states (synchronized slow II and synchronized fast) (top) and periods of full eye closure (bottom) in one monkey dataset. Vertical bars represent 1s time segments. **(E)** Pearson correlation coefficient calculated between the time course of eyes closure and the time courses of different cortical states.

Having identified different cortical states and their statistical properties, we next studied how synchronization in the visual cortex evolved over time in the cat, where we had acquired continuous recordings up to one-hour duration. In general, the cortex not only fluctuated between the different states at a time scale of seconds, but the probability to find synchronized or desynchronized states also changed at much longer time scales (Fig 6A). These fluctuations appeared at very slow timescale (<0.01 Hz) with a peak at ~ 0.001–0.002 Hz (Fig 6B), indicating the presence of ultra-slow dynamics during anesthesia with long periods of predominant synchronization or desynchronization.

In the monkey, short periods of synchronization (several seconds) alternated with longer temporal epochs of desynchronization (Fig 1A). As the monkey was resting in the dark, we hypothesized that state changes coincided with the monkey becoming drowsy and closing the eyes. Eye tracking analysis revealed that the animal indeed closed the eyes during extended periods of time, on average 8 ± 4.8s (Fig 6C). These epochs of eye closure were associated with the cortex shifting to synchronized states (Fig 6D). In particular the occurrence of fast (p < 0.001) and slower synchronized (SynSlow II) states (p < 0.001) was highly correlated with the closed eye condition, while desynchronization showed significant anticorrelation (p < 0.001) (Fig 6E). This confirms that the level of spike synchronization and hence criticality is closely related to the vigilance state of the animal.

### Statistics of the joint neuronal activity patterns

**Fig 7:**
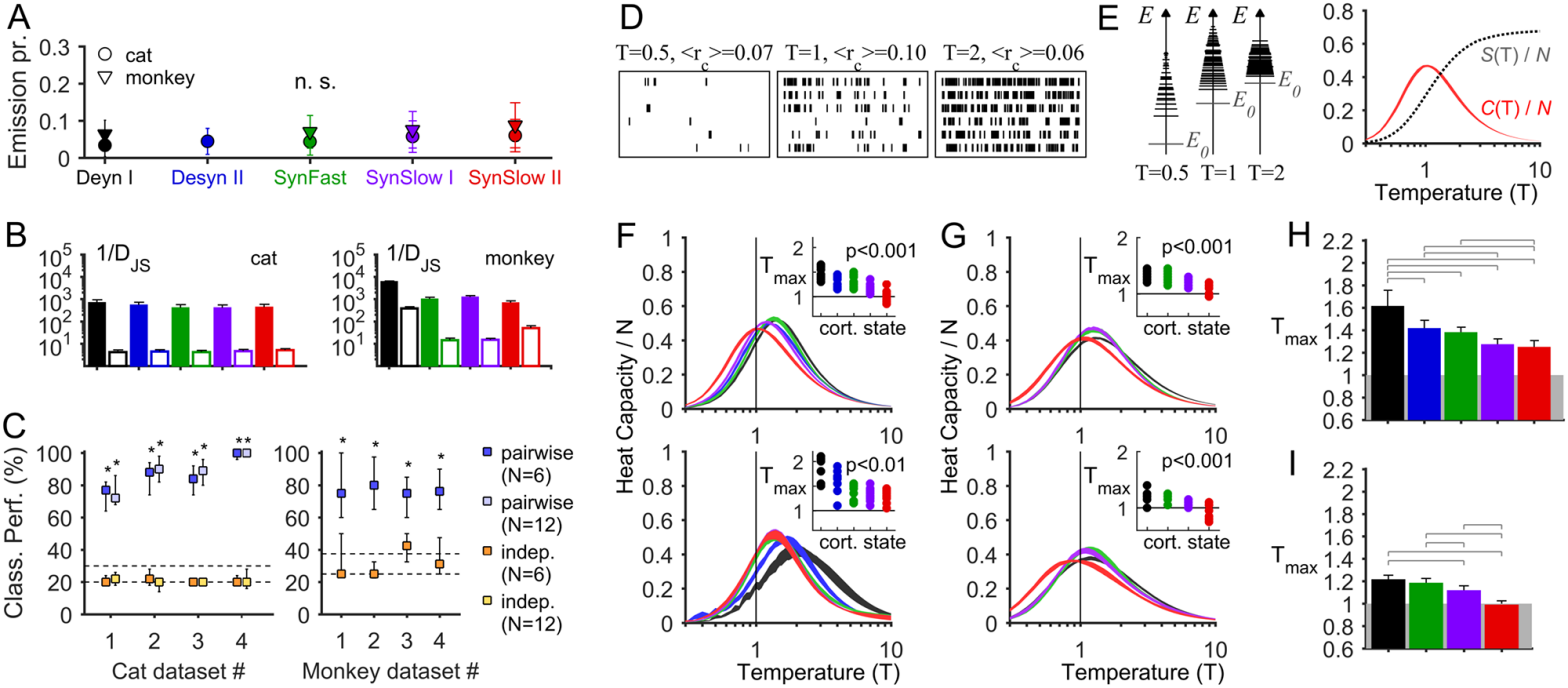
Maximum entropy models (MEMs) of different cortical state. **(A)** Probability that an electrode site *i* has σ_*i*_ = +1 (emission probability) using the bin sizes indicated in Table 2 (error bars indicate SEM). The emission probabilities do not significantly depend on the cortical state (p = 0.092 for cat data, p = 0.058 for monkey data, rm-ANOVA). **(B)** Goodness-of-fit (1/*D*_*JS*_) of pairwise-MEMs (filled bars) and independent-MEMs (open bars) for each cortical state (averaged over the 10 groups of *N* signals and all datasets; error bars indicate SEM). **(C)** Prediction of the cortical state using pairwise- and independent-MEMs. The percentage of correct classifications is shown for each cat (*left*) and monkey (*right*) dataset. Squares indicate the medians and error bars delineate the 5–95th percentiles of the classification performance. Dash lines: mean and 95th percentile of the number of correct classifications expected by chance. *: significant classification performances (p < 0.05). **(D–E)** Effect of changing the temperature parameter T on the model activity. The MEM in this example was estimated using the activity of a neural ensemble of the cat in the SynSlow II state. **D**: model activity (1000 steps are shown out of 10^6^ steps). At low temperature (T = 0.5) the activity is sparse and correlations are low (*<r*_*c*_*>* = 0.07); at high temperature (T = 2) the activity is dense and random and correlations are low (*<r*_*c*_*>* = 0.06); for an intermediate temperature (T = 1) the activity is more patterned and correlations are higher (*<r*_*c*_*>* = 0.10). **E**: *Left*, occupied energy levels. The size of the horizontal lines is proportional to log(*n*_*E*_), where *n*_*E*_ is the number of patterns that have the energy *E*. *Right*, the entropy *S*(T) increases with T and the heat capacity *C*(T) peaks at a given temperature T_max_ (for this particular example neural ensemble T_max_ = 1). **(F–G)** Heat capacity as a function of the temperature parameter (T), for each cortical state, for two example anesthetized cat datasets **(F)** and two example awake monkey datasets **(G)** (10 random choices of groups of *N* signals were used; trace thickness indicates SEM). *Inset*: Peak temperature (T_max_) for each neuronal ensemble and for each cortical state. T_max_ significantly depends on cortical state (p: p-value, rm-ANOVA). **(H–I)** T_max_ averaged over all cat datasets **(H)** and over all monkey datasets **(I)** for each cortical state (rm-ANOVA: H: *F*_4,156_ = 25.03, p < 0.001, *ε* = 0.34; I: *F*_3,117_ = 59.60, p<0.001, *ε* = 0.90). Error bars indicate SEM. Horizontal bars: significant differences (p < 0.05) of subsequent Bonferroni's test for multiple comparisons. In A–I the size of the models was *N* = 6.

Next, we studied the patterns of the ensemble spiking activity from *N* recording sites during the different cortical states and examined their statistics in the framework of statistical mechanics. This enabled us to have further physical characterization of the different cortical states in terms of criticality. Time was discretized in non-overlapping bins. In a time bin Δ*t*, the state of the neural ensemble is described by a binary pattern $\vec{\sigma }=\left[\sigma_{1},\;\sigma_{2},\ldots ,\;\sigma_{N}\right]$, where σ_*i*_ = +1 if the *i*-th electrode site recorded one or more spikes (activation) and σ_*i*_ = −1 otherwise (quiescence). Δ*t* was chosen to obtain homogeneous averaged activation rates across datasets and cortical states (Fig 7A, see also Table 2). We aimed at obtaining the probability distribution $P\left(\vec{\sigma }\right)$ over the 2^*N*^ possible binary patterns. For this, we used a Maximum entropy model (MEM) to find $P\left(\vec{\sigma }\right)$ by maximizing its entropy under the constraint that the activation rates (<σ_*i*_>) and the pairwise correlations (<σ_*i*_σ_*j*_>) are preserved (see Methods). The resulting distribution is $P\left(\vec{\sigma }\right)\propto \;e^{-E\left(\vec{\sigma }\right)}$, where $E\left(\vec{\sigma }\right)$ is the energy of the pattern $\vec{\sigma }$, and $E\left(\vec{\sigma }\right)=-\sum {}_{i}^{N}\left[h_{i}\sigma_{i}+\frac{1}{2}\sum {}_{j}^{N}J_{ij}\sigma_{i}\sigma_{j}\right]$, where *h*_*i*_ represents the intrinsic tendency of site *i* towards activation and *J*_*ij*_ represents the effective interaction between sites *i* and *j* (see Methods). Note that energies are proportional to the patterns’ minus log probabilities, or “surprise”. The model parameters **Ω** = {***h***; ***J***} were estimated from the data using a gradient descent algorithm (see Methods). We constructed pairwise-MEMs for each dataset and each cortical state separately and analyzed the obtained models. Here, we present analyses of the joint activity of *N* = 6 electrodes for both cat and monkey datasets—but, as shown below, we found consistent results for models of larger size for the cat datasets, for which larger ensembles were recorded (see Table 2).

**Table 2:** Total number of active electrode sites (*n*), number of observed binary patterns (*L*), and bin size (*b*) for each cortical state and each dataset.

Cortical State:	Cat								Monkey							
	Dataset 1 (*n* = 27)		Dataset 2 (*n* = 25)		Dataset 3 (*n* = 22)		Dataset 4 (*n* = 12)		Dataset 1 (*n* = 6)		Dataset 2 (*n* = 9)		Dataset 3 (*n* = 10)		Dataset 4 (*n* = 10)	
	*L*	*b*	*L*	*b*	*L*	*b*	*L*	*b*	*L*	*B*	*L*	*b*	*L*	*b*	*L*	*b*
**Desyn I**	38,379	50	70,248	50	77,395	50	77,395	50	34,700	10	51,499	10	36,996	10	54,199	10
**Desyn II**	21,259	50	25,080	50	25,080	50	10,440	50	--	--	--	--	--	--	--	--
**SynFast**	6,860	50	14,360	50	14,360	50	5,020	50	6,191	20	1,300	20	4,350	20	650	20
**SynSlow I**	7,640	50	2,880	50	2,880	50	4,280	50	3,850	20	2,200	20	2,150	20	1,750	20
**SynSlow II**	3,860	50	7,380	50	7,380	50	22,860	50	2,600	20	750	20	5,000	20	500	20

We first evaluated the importance of correlations by comparing the performance of the pairwise-MEMs to the performance of independent-MEMs for which only the activation rates (<σ_*i*_>) are preserved (i.e., only ***h*** is optimized). Specifically, we compared the prediction and the empirical estimation of the probabilities of spiking patterns in each cortical state. We found that taking into account the pairwise correlations improves the agreement with the data by 1–2 orders of magnitude (Fig 7B). Second, we investigated how specific the different MEM parameters are to the different cortical states by measuring the ability of the model to predict the cortical state from neural ensemble activity. We used a jackknife cross-validation procedure, consisting of first estimating the MEM parameters on a subset of the available data for each cortical state and then using the learned MEM parameters to classify the remaining data (Methods). We found that, on average, pairwise-MEMs predicted the cortical state of the cat data with 75.8–98.7% and 74.8–99.7% accuracy for *N* = 6 and *N* = 12, respectively, and of the monkey data with 73.1–83.4% accuracy for *N* = 6 (Fig 7C). However, when using independent-MEMs the performance hardly exceeded chance level. These results confirm that the MEM parameters, specifically the interaction couplings *J*_ij_, tightly relate to the cortical state.

Next, we further studied the learned MEMs to obtained relevant features of the collective dynamics in each cortical state. One important quantity is the heat capacity, *C*, that relates to how the distribution of energies changes as a function of a parameter T, analogous to the temperature in statistical physics and equivalent to scaling all model parameters as **Ω** → **Ω**/T (see Methods). Specifically, *C* is given by *C*(**Ω**,T) = var[*E*]/T^2^. The “temperature” T controls the level of disorder and its effect can be understood by examining the energy levels {*E*} that are accessible to the system. At low temperatures the system is predominantly silent, it accesses few and separated excited states, it is relatively ordered (its entropy *S* is low), and, since interactions dominate, the system scarcely fluctuates, leading to weak correlations (Fig 7D and 7E). At high temperatures the system has a high probability of occupying the excited states, which are barely separated, making it easy to fluctuate among them, thus increasing the disorder and decreasing the correlations (fluctuations dominate over interactions, effectively decorrelating the system). As for low temperatures, high temperatures lead to a low *C*. However, for a particular temperature, T_max_, a large range of energies is accessible to the system, leading to a maximal *C*, and, moreover, the balance of fluctuations and interactions leads to a maximal mean correlation. This is the expected behavior close to a critical point where both order and disorder coexist [59]. In conclusion, a maximal heat capacity close to the operating point (i.e., T_max_ = 1, corresponding to the model of the real data) suggests that the system is likely to be close to a critical state.

We calculated the heat capacity *C* as a function of T for each cortical state (Fig 7F–7I). We found that the peak of *C* is located at different temperatures for different cortical states: in all 4 cat datasets and in all 4 monkey datasets the temperature T_max_ at which *C* is maximal is significantly cortical-state-dependent (p < 0.01, one-way rm-ANOVA, Fig 7F and 7G) and is the highest for the most desynchronized cortical state and the lowest (approaching T_max_ ≈ 1) for the most synchronized cortical state (Fig 7H and 7I).

**Fig 8:**
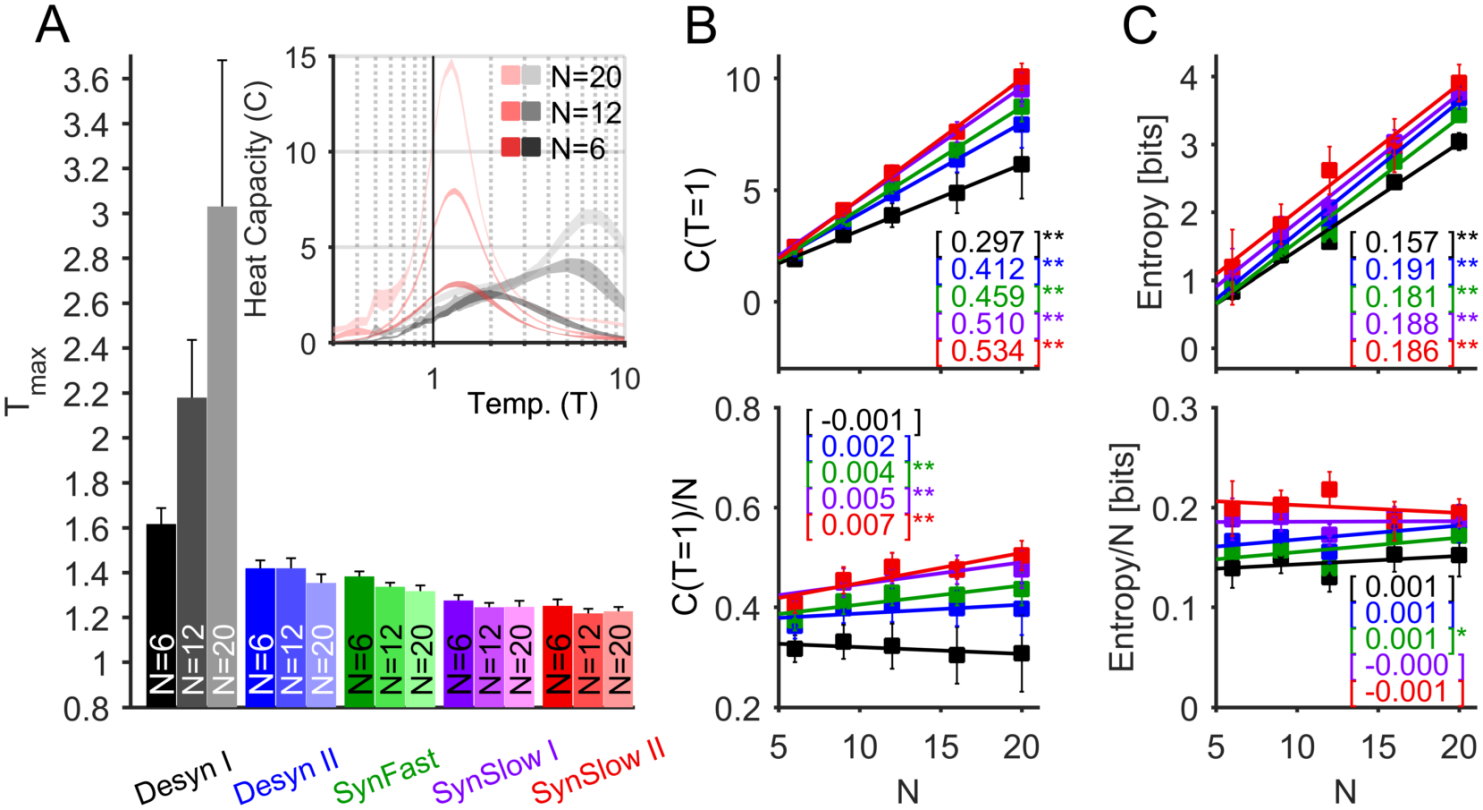
Heat capacity and entropy as a function of ensemble size. **(A)** Peak temperature (T_max_) averaged across anesthetized cat datasets for each cortical state and for ensembles of different sizes *N*. This analysis was restricted to cat datasets, for which more electrodes and longer recordings were available (datasets #1–4 for *N* ≤ 12 and datasets #1–3 for *N >* 12). *Inset*: heat capacity functions for Desyn I (*gray scale*) and SynSlow II (*red scale*) for an example cat dataset. **(B)** Magnitude of the heat capacity, *C* (*top*), and specific heat capacity, *C/N* (*bottom*), evaluated at T = 1 for different ensemble sizes *N*. For all tested *N*, the heat capacity was significantly cortical-state-dependent (p < 0.05, rm-ANOVA). **(C)** Entropy (*top*) and specific entropy (*bottom*) as a function of *N*. For all tested *N*, the entropy was significantly cortical-state-dependent (p < 0.05, rm-ANOVA). In both (B) and (C) lines indicate least-squares linear fit, for which the slopes are showed in the brackets and the asterisks indicate slopes significantly different from zero (*: p < 0.05, **: p < 0.01), and error bars indicate SEM.

We further studied the collective dynamics for different ensemble sizes *N*. For increasing *N* the heat capacity of the data grows and the peak temperature decreases for all cortical states, except for the most desynchronized state for which T_max_ increases (Fig 8A and 8B). Notably, the heat capacity of the data significantly grows even when it is normalized by *N*, as expected close to a critical point [59], for all but the desynchronized states (Fig 8B). Lastly, as a consequence of the relation between entropy and *C* (see Methods), the entropy of the spiking patterns significantly depends on the cortical state (p < 0.05, rm-ANOVA) and it is higher in synchronized states (Fig 8C). The mean entropy differences between SynSlow II and Desyn I is *ΔS* = 0.428 bits (average over all cat and monkey datasets, for *N* = 6); this is a substantial change, since this implies that the pattern repertoire is reduced 2^*NΔS*^ ≈ 6–fold from the synchronized to desynchronized states. Altogether these results show that synchronized cortical states are closer to a maximum possible range of surprise (energies) and further suggest that synchronized cortical states are close to criticality.

### Spiking model and sub-sampling analysis

**Fig 9:**
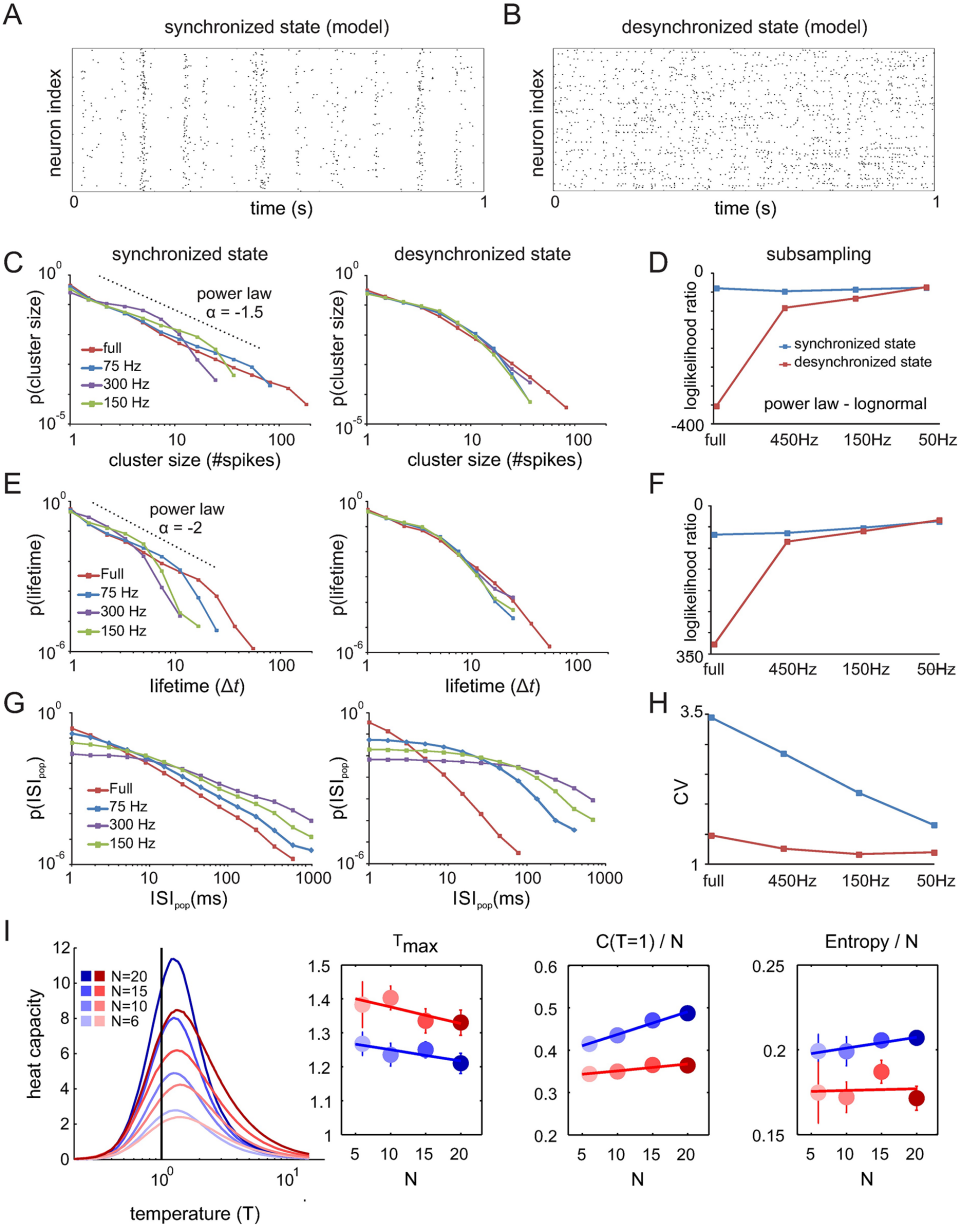
Sub-sampling analysis of a spiking neuronal model. **(A-B)** Model spike rasters of synchronized and desynchronized states. **(C)** Cluster size distributions of synchronized and desynchronized model activity. **(D)** Loglikelihood ratios of fitted power law and lognormal distributions fitted to the cluster size distributions for different levels of sub-sampling. **(E)** Lifetime distributions for synchronized and desynchronized model dynamics. **(F)** Same as in (D) for lifetime distributions. **(G-H)** Inter-spike interval distributions of population spike trains (ISI_pop_) and corresponding coefficients of variation for three different degrees of sub-sampling. **(I)** Heat capacity as a function of the temperature parameter (T), for each dynamical regime of the spiking network (*blue*: critical, *red*: subcritical). Twenty random choices of groups of *N* model neurons were used; error bars indicate SEM. The insets show: the peak temperature (T_max_) as a function of *N*, the amplitude of the heat capacity normalized by *N*, and the entropy per neuron in both dynamical regimes. Lines indicate least-squares linear fits.

In the following, we evaluated the impact of using a small sample of recorded neurons on our analyses. Sub-sampling has been shown to underestimate neuronal correlations [60] and can lead to a breakdown of power law relationships [21,28,34]. In contrast, absence of power laws may also be due to genuine deviations from critical dynamics as we show above. To test how sub-sampling affects the discrimination between cortical states and their critical properties, we modeled a realistic spiking neuron network (see Methods) which is capable of generating population dynamics that closely resemble those seen in physiological recordings [35,53,61,62]. Depending on the overall drive, the network can exhibit irregular bouts of synchronized bursts (synchronous irregular state [61], SI, low input) or largely desynchronized activity (asynchronous irregular state [61], AI, high input), akin to the physiological states shown in this study (Fig 9A and 9B). In accordance with the predictions from criticality and similar to the presented *in vivo* data, synchronized activity in the fully sampled network displays power laws in cluster size distributions with an exponent close to -1.5, power laws in lifetime distributions with exponents approaching -2 and CV values of inter-spike interval distributions >>1 (Fig 9C, 9E and 9G). In contrast, desynchronized states in the full model created clearly curved distributions with loglikelihood ratios being much more negative than in synchronized states (Fig 9D and 9F). In addition, CV values of the inter-spike interval distribution were close to 1, as expected from a Poisson process. Finally, we investigated whether the simulated neuronal avalanches produced self-similar dynamics (shape collapse) as predicted by criticality theory (S3G–S3J Fig). We found that, in the synchronized state, the spiking network produces avalanches whith a temporal evolution that could be rescaled, while desynchronized model activity patterns did not collapse suggesting a departure from critical dynamics.

Next, we sub-sampled the model by randomly removing neurons (50 times) such that the cut off in power laws seen in the cluster size distributions resembled those seen in the cat (cutoff: ~60 spikes; population rate: 450 Hz) and monkey recording (cutoff: ~20 spikes; population rate: 150 Hz). In both cases, LLRs of cluster size and lifetime distributions as well as CV values of inter-spike interval distributions remained distinguishable from desynchronized activity sub-sampled with the same degree. With more severe sub-sampling (population rate: 50 spikes) cluster size and lifetime distributions could not be delineated by loglikelihood ratios anymore. Only CV values of interspike interval distributions remained sensitive enough to separate synchronized and desynchronized states at this level of sub-sampling. In conclusion, these results demonstrate that if synchronization levels of the population dynamics remain sufficiently strong, cluster size and lifetime distributions remain sensitive to differences in dynamical states despite heavy sub-sampling.

Sub-sampling could be also an issue when analyzing the spiking activity with the MEM. We thus studied the behavior of the heat capacity and entropy functions obtained for different ensemble sizes *N* for the spiking model activity in the subcritical and critical regimes. As in our previous analyses on the empirical data, we constructed MEMs of size *N* = 6–20 by randomly selecting *N* model neurons. The bin sizes were chosen to obtain the same averaged emission probability as in the empirical data (equal to 0.048). We next used the learned models to calculate the heat capacity curves given by estimating the variance of the energies (*E*) from Monte Carlo simulations (5.10^5^ steps) for different temperature parameters T. As shown in Fig 9I, the heat capacity behaved as expected for the two different network regimes, even for small neuronal ensembles of *N* = 6. The peak temperature T_max_ was significantly different between the two dynamical regimes (p < 0.001, t-test), it was the lowest for the critical regime and approached 1 for increasing *N*. Furthermore, the normalized heat capacity (*C/N*) significantly increased with *N* for the critical regime (slope = 0.005, p < 0.001) but remained constant in the subcritical regime (slope = 0.002, p > 0.05). Finally, the resulting entropy was higher in the critical regime than in the subcritical regime (p < 0.01, t-test). Note also that the values of the heat capacity and the entropy were very similar to the ones obtained using the empirical data. We conclude that MEMs constructed using a limited number of neurons effectively discriminate between the different network regimes.

Lastly, we also investigated whether the statistical differences in our data are due to artificially dividing critical dynamics into different states. To this end we divided the simulated critical model dynamics into different synchronization regimes based on the Fano factor of the population spike train (see Supplementary Material), which shows differences across states in the experimental spiking data (S4A–S4C Fig). In contrast to the recordings, collapse and power laws, albeit with smaller cutoff sizes, were preserved during periods of reduced levels of population synchronization and firing rate in the model SI state (S4D–S4F Fig). These results are expected since the self-similarity in the model creates dynamics with statistics that are immune to changes of observation scale, in this case limiting analysis to less synchronized activity. They also indicate that the differences in power law statistics in the recordings reflect genuine state changes and are not due to artificial separation of a critical dynamical regime into different states.

## Discussion

We have shown that neuronal dynamics in the primary visual cortex during anesthesia and in the awake state transiently unfold in a critical state. In our study, the cortex underwent continuous state transitions with varying levels of LFP power in slow frequency bands and overall spike synchronization. Furthermore, the degree of synchronization was linked to the degree of criticality as measured by power laws and heat capacities calculated using collective spiking activity. Notably, the extent of critical dynamics could be predicted from the amount of fluctuations in local field potentials and the vigilance state of the animal, associating states of prolonged eye closure and putative drowsiness with higher levels of criticality.

These findings are in contrast to previously reported invariant presence [21] or absence of critical features in spiking activity [31,32,51]. In the latter studies, invariant deviations from power law were found across several species, brain areas and stages of the sleep wake cycle after averaging criticality measures across entire datasets. However, after having separated statistics for various cortical states, we have shown that the cortex is indeed capable of transiently generating dynamics that come close to criticality. Moreover, our analyses using the MEM confirm that cortical states have consistently different second-order statistics and demonstrate that cortical states are truly different physical states in the sense of statistical physics. Our results are in line with a previous study which reported fluctuations of criticality measures in spiking activity recorded in the barrel cortex of rats under urethane anesthesia [44]. We extended this finding to the primary visual cortex of anesthetized cat and awake monkey and showed in the latter condition that the vigilance state as measured by prolonged periods of eye closure can predict a switch to dynamics closer to criticality. It has been known for a long time that EEG synchronization in the visual cortex is stronger when eyes are closed as compared to when they are open [63]. Here, we link the “closed eye” state not only with higher synchronization in LFP and spiking activity, but also with dynamical changes towards a critical state. Moreover, we found that the level of synchronization during isoflurane anesthesia in the cat can vary at very slow time scales which is in accordance with a recent report in opioid anesthetized monkeys [64].

Another recent study, however, reported conflicting results obtained with calcium imaging in the frontal cortex of head-fixed awake and isoflurane anesthetized rats [22]. Firing rates and correlations remained stable within each vigilance state, while power laws were only found during waking. In contrast, we found largely subcritical distributions during the awake state during the eyes open condition, which were replaced by more critical distributions during the closed eye condition. In addition, correlations during anesthesia were not stationary in our study despite having used the same anesthetic agent, similar to other studies [38,44,64]. These discrepancies may originate in different levels of anesthesia (0.8% isoflurane concentration in this study, 1–2% in [22] potentially influencing the appearance of state fluctuations, temporal resolution of spike recordings (fast electrophysiological recordings vs. slow calcium imaging) or recording site (primary visual cortex vs. frontal cortex). Further research needs to be undertaken to clarify these different results.

Our findings of a tight link between state changes are difficult to reconcile with the sub-sampling hypothesis of criticality which attributes subcritical distributions in spiking activity to the inevitable sub-sampling of neuronal discharges in empirical data and has been used to explain absence of criticality [20,21,28,34]. This susceptibility to sub-sampling has rendered spiking activity a less likely candidate for finding robust features of criticality [11], even though spiking activity is the backbone of neuronal avalanches in the brain. In contrast, theoretical studies have demonstrated that neural networks can display different collective behaviors, such as asynchronous irregular dynamics, in which excitatory avalanches are precluded by fast inhibition and strong external drive, or synchronized bursting activity, which can be accompanied by statistics expected from a critical state [32,53,62,65]. As predicted from these models, previous experimental work has indeed found similar dynamical transitions of spiking activity [37–41,66]. The spiking neuronal network model used in the current study was also able to reproduce different states with statistics similar to our *in vivo* results, showing synchronized states with signs of criticality and subcritical desynchronized states as a function of external drive. Importantly, differences between distinct states in the model were detectable in criticality statistics even with strong sub-sampling that mimicked the cutoff of power laws seen in the monkey and cat recordings, providing support that the sampling in our experiment was sufficient to observe deviations from criticality in our data. Moreover, the MEM model revealed robust differences in criticality between states requiring only a small sample of neurons (~10). In conclusion, we provide evidence that criticality statistics vary despite invariant spatial sampling of neuronal discharges and can show near critical features, when the cortex is engaged in synchronized population dynamics.

The existence of different cortical states and corresponding levels of criticality raise the question of their functional significance. Traditionally, the larger number of possible neuronal patterns provided by the complex dynamics in a critical state has been suggested to optimize a number of cortical functions for which some evidence has been found experimentally [9,67].

However, recent experimental data suggest that active processing may actively desynchronize spiking activity in the cortex [35,39–41,43,68] through neuromodulatory or glutamatergic signals [35,69], in a way similar to the desynchronized and subcritical dynamics described in this study. This desynchronization reduces response variability and enables a reliable representation of stimuli, in contrast to synchronized states [70–72]. In addition, desynchronized activity may be necessary to establish precise and unambiguous communication between areas [73], possibly embedded within gamma oscillations [74,75] that are more prevalent during cortical desynchronization [76,77]. Consistent with this line of reasoning, task related focused attention was found to trigger subcritical dynamics in EEG recordings of large cortical networks of humans [50]. The same study, however, found near critical dynamics during resting state arguing in favor of criticality when cognitive demands require exploration of different network states. Similarly, we observed that collective dynamics during synchronized states approach the maximal range of surprise (heat capacity), indicating that the cortical system might be spontaneously exploring its dynamical repertoire (i.e., the system visits a set of configurations, or patterns, more diverse in terms of associated energies; Fig 7). Another hypothesis considers desynchronized states as quasi-critical, maintaining optimization of computational functions in the absence of strict criticality [78]. Finally, our results are consistent with recent studies showing that hierarchical connectivity of neural networks can enlarge the region where critical-like behavior is observed, the so-called Griffith phase [79–81]. In this phase, rare occurrence of local order can be observed even though the system is globally in the disordered phase, thus making it a candidate mechanism for the observed transitions between cortical states. Further studies are required to clarify the precise cognitive role of critical dynamics in the brain.

In conclusion, we have shown that the cortex can transiently approach criticality during anesthesia and awake states in the primary visual cortex. These fluctuations of criticality can be predicted from more global variables such as the LFP and also reflect different vigilance states. Our results suggest that the cortex evolved mechanisms to synchronize and desynchronize its activity according to computational needs, thereby continuously switching between critical and more subcritical dynamics.

## Materials and methods

### Preparation

Recordings were obtained from four anesthetized (isoflurane) and paralzyed cats (three females, one male), and one awake monkey (male). The isolfurane concentration was kept at a constant value of ~0.8%. Experimental protocols on behaving monkeys have been approved by the Marseille Ethical Committee in Neuroscience (approval #A10/01/13, official national registration #71-French Ministry of Research). The animals used in the experiments were bred in the Central CNRS Animal Care (French Agriculture Ministry Authorization: B91-272-105) under required veterinary and National Ethical Committee supervision.

### Recording

We inserted 32 channel silicon-based micro-electrode arrays (four shanks with eight electrode contacts each, distance between electrode contacts: 400 μm, electrode contact impedance: 0.3–0.5 MΩ at 1000 Hz, shank length: 3mm, Neuronexus Technologies, Ann Arbor, USA) into area 17 of four cats (one array per cat) and chronically implanted a Utah array (Blackrock Microsystems, Salt Lake City, UT, USA) into the primary visual cortex (near-foveal retinotopic region) of an adult macaque monkey (macaca mulatta—10kg). The Utah array was composed of 96, 1 mm long, electrodes arranged in a 10 x10 matrix with an inter-electrode distance of 400 microns. In both preparations, continuous spontaneous multiunit activity (sampling frequency: 30 kHz,) and local field potentials (LFP, sampling frequency: 1 kHz) were recorded with a Cerebus acquisition system (Blackrock Microsystems, Salt Lake City, UT, USA). For each cat we acquired one dataset lasting between 3900 and 6000 seconds, while four datasets of 600 seconds each were obtained in the monkey on four different days. The monkey was sitting in the complete dark, head fixated. For subsequent analysis the LFP was filtered between 1 and 100 Hz. Spikes were detected by manually setting a one-sided threshold that would result in a signal to noise ratio larger than ~2. Action potential waveforms were sorted and separated from remaining noise using the T-EM clustering algorithm and manual cluster cutting (Offline Sorter, Plexon Inc, Dallas, USA). In the cat, simultaneous recordings of spiking activity were extracted from an average of 21.5±6.7 channel sites and in the monkey 8.75±1.9 channels sites. Note that no attention was paid to extract single units, as during later analysis spikes from each electrode were pooled together into one population spike train. The mean firing rate per site ranged between 0.61 Hz and 1.44 Hz (1.01±0.38) in the cat and between 4.95 Hz to 7.32 Hz (6.31±1.08) in the monkey recordings. The rates for the entire recordings were 20.64±7.9 Hz for the cat data and 54.24±10.57 Hz for the monkey. The cat recordings were performed in uniform scotopic illumination level, while any source of light was eliminated during the monkey recordings. During three recording sessions in the monkey we obtained eye tracking data using the Eyelink 1000 system (SR research, sampling frequency 1000Hz).

### Separation of cortical states

Cortical states and degrees of synchronization of neuronal activity can switch at a time scale of seconds [35]. In order to capture this fast dynamics a moving temporal window of one second was chosen to analyze different synchronization levels in cortical activity. We based the separation of cortical states on differences in the frequency composition of the LFP between the one second segments. To this end we computed power spectra for each channel of a given segment with the multitaper method (see http://chronux.org) and averaged across all channels to obtain one spectrum per segment. Next, we split the power spectrum into 1 Hz frequency bins between 1 and 100 Hz and fed these 100 values as variables into a principal component analysis (PCA). As a result, we reduced the power spectrum of each segment to its first three principal components and each segment was represented by its position in a three dimensional PCA state space. In order to find segments with similar power spectra, we applied a k-means clustering algorithm with a different number of clusters (2–10 clusters) after normalization of each principal component to a value of 1. To determine the optimal number of clusters we validated the clustering results with the Dunn index [82] according to the following formula:

$$DI=\underset{1\leq i\leq n}{\text{min}}\left\{\underset{1\leq j\leq n,i\neq j}{\text{min}}\left\{\frac{d\left(i,j\right)}{\underset{1<k<n}{\text{max}}d^{\prime }\left(k\right)}\right\}\right\},$$

with distance *d(i*, *j)* between the centers of clusters *i* and *j* and distance *d'(k)* between the center and elements of cluster *k*. This equation evaluates the compactness of clusters by calculating the ratio between the minimal distance between clusters to the maximal distance within clusters. The Dunn index was computed for each dataset and then averaged separately across all cat datasets and all monkey datasets. The Dunn index reaches a maximum at the optimal cluster number and we identified five clusters for each cat dataset and four clusters for each monkey dataset (Fig 1C and 1D). The clusters of each recording were consistently color coded according to their position in the state space, such that the clusters were comparable across datasets and species. The different clusters were associated with different cortical states. All the segments of a cluster were concatenated and used as separate datasets in subsequent analysis.

### Correlation analysis

To investigate the correlation characteristics of the recorded neuronal population, we collated together the spiking activity of all channels into one population spike train. Correlations were assessed by computing the auto-correlation histograms (ACH). An ACH was calculated for each 1 second segment and subsequently averaged across all segments of a cortical state. We also subtracted a shift predictor that was calculated from the average of 100 randomly shuffled surrogates with the same firing rate to normalize the baseline for each segment. All ACHs were normalized to a center peak of one and quantified based on the peak amplitude (i.e. after removal of the peak at zero time lag) and the integral between the ACH curve and the baseline for time lags between -250 and 250ms. In order to allow a comparison between different datasets and species, these measures were normalized such that the cortical state with the maximum value for each dataset was set arbitrarily to one.

We also estimated the correlation between spiking activity and the LFP by constructing spike triggered averages (STA) for each cortical state of a given dataset. The STAs of all clusters and datasets showed a negative deflection. LFP was z-scored and STAs were quantified by calculating the amplitude of the deflection peak and the integral of the negative deflection with respect to the zero crossings. Like for the ACH, these two measures were normalized to the cortical state with the maximum value which was set to one.

### Neuronal avalanche analysis

The spiking data were binned with bin-size Δt and event clusters were defined as groups of consecutive bins each containing a number of spikes above a predefined threshold. This threshold was varied between 1–5 spikes in the cat and 1–3 spikes in the monkey. In both cases the upper bound was given by the threshold that yielded enough avalanches to fit distributions (see below). The first and last avalanches of an analyzed time window were discarded, if they were not preceded or followed by a bin with a number of spikes below the threshold, respectively. The cluster size was equal to the number of events within a cluster. In accordance with previous studies [13,21], Δt was chosen as the average inter-spike interval of the population spike trains (also denoted as ISI_pop_, Table 1) which reflects a compromise between spurious concatenation of small clusters (large Δt) and separation of larger clusters (small Δt). The lifetime of an avalanche was defined as the number of bins spanning an avalanche. Distributions of cluster sizes and lifetimes were plotted in log-log coordinates and further analyzed.

In order to statistically characterize the distribution of cluster sizes s, we first fitted a power law distribution *P*(*s*, *α*) ~ *Cs*^*α*^, with power law exponent *α*. Since on visual inspection size distributions in desynchronized states clearly deviated from a power law, we also fitted lognormal distributions with the following probability density function: $N\left(s,\mu ,\sigma \right)=\frac{1}{\text{s}\sigma \surd 2\pi }\text{exp}\left(-\frac{{\left(\text{ln}s-\;\mu \right)}^{2}}{2\sigma^{2}}\right)$; *σ* > 0, *μ* ≥ 0 with scale parameter *σ* and location parameter *μ*. Depending on *σ*, this distribution can assume heavy tails close to a power law or lighter tails. We then compared the fits to both distribution by calculating the loglikelihood ratio and a corresponding p-value determining the significance of model fit differences [22,52]. In our study, positive LLR values indicated superior power law fits, while negative values favored lognormal distributions. All fits were performed using the Python toolbox for analysis of heavy-tailed distributions [83]. Parameters were calculated for the entire distribution. The same fitting procedures were applied to lifetime distributions.

#### Maximum entropy models (MEMs)

We further studied the patterns of the ensemble spiking activity from *N* recording sites during the different cortical states in the framework of statistical mechanics. The ensemble activity was binarized in non-overlapping time bins of 50 ms for anaesthetized cat data, and 10 or 20 ms for awake monkey data (different time bins were chosen to compensate for the different averaged activation rates between datasets and cortical states; see Table 2 and Fig 7A). In a time bin Δ*t*, a single electrode site *i* either did (σ_*i*_ = +1) or did not (σ_*i*_ = −1) generate one or more spikes, thus, the state of the neural ensemble is described by a binary pattern $\vec{\sigma }=\left[\sigma_{1},\sigma_{2},\ldots ,\sigma_{N}\right]$. We used a Maximum entropy model (MEM) to estimate the distribution $P\left(\vec{\sigma }\right)$ of each of the 2^*N*^ possible patterns, i.e., we estimated $P\left(\vec{\sigma }\right)$ by maximizing its entropy under the constraint that some empirical statistics are preserved. A pairwise-MEM provides a solution under the constraint that the activation rates (<σ_*i*_>) and the pairwise correlations (<σ_*i*_σ_*j*_>) are preserved. It is known that the maximum entropy distribution *P* that is consistent with these expectation values is given by [59,84]:

$$P\left(\vec{\sigma }\right)=\frac{e^{-E\left(\;\vec{\sigma }\right)}}{Z},$$

where the normalization factor $Z=\Sigma_{s=1}^{2^{N}}\text{exp}\left[E\left({\vec{\sigma }}_{s}\right)\right]$ is the partition function and $E\left({\vec{\sigma }}_{s}\right)$ is the energy of the pattern *s*, with *s* ∊ {1,…,2^*N*^}, and *E* is given by:

$$E\left(\vec{\sigma }\right)=-\sum_{i=1}^{N}h_{i}\sigma_{i}-\frac{1}{2}\sum_{i=1}^{N}\sum_{j=1}^{N}J_{ij}\sigma_{i}\sigma_{j},$$

where, *h*_*i*_ represents the intrinsic tendency of site *i* towards activation (σ_*i*_ = +1) or silence (σ_*i*_ = −1) and *J*_*ij*_ represents the effective interaction between sites *i* and *j*. Note that the energy represents the patterns’ minus log probabilities, log *P*, or surprise, plus the constant log *Z*. The estimation of the model parameters ***h*** and ***J*** was achieved through a gradient descent algorithm (see below). Once we know the parameters **Ω** = {***h***; ***J***} the expected probability of any pattern is known, by first calculating the energy associated to the pattern and then computing *P*. For each recording session, we constructed models for each cortical state separately by, first, randomly selecting *N* signals, second, concatenating the subset of all binarized patterns belonging to the same cortical state, and, then, running the parameter learning procedure for each subset of patterns (Table 2 summarizes the characteristics of the data). We did this for a total of *Q* = 10 random choices of ensembles of *N* signals.

#### Estimation of MEM parameters

The MEM parameters *h*_*i*_ and *J*_*ij*_ were iteratively adjusted to minimize the absolute difference between the empirical activation rates (<σ_*i*_>) and correlations (<σ_*i*_σ_*j*_>) and those (<σ_*i*_>_model_, <σ_*i*_σ_*j*_>_model_) predicted by the model through Monte Carlo simulations. Specifically, each iteration is given by: $h_{i}^{new}=h_{i}^{old}-\alpha \left({\langle \sigma_{i}\rangle }_{\text{model}}-\langle \sigma_{i}\rangle \right)$, and $J_{ij}^{new}=J_{ij}^{old}-\alpha \left({\langle \sigma_{i}\sigma_{j}\rangle }_{\text{model}}-\langle \sigma_{i}\sigma_{j}\rangle \right)$, where *α* is the learning rate (α = 0.1). In our study, we stopped the re-estimations once the differences between the empirical and model values are less than a tolerance threshold (0.005) or if this tolerance was not reached within a maximum number of iterations (100).

*MEM goodness-of-fit*. The goodness-of-fit of the MEMs was evaluated using the Jensen–Shannon divergence (*D*_*JS*_) between the probability distribution of the empirical and model binary patterns [85]. *D*_*JS*_ is a symmetric version of the Kullback-Leibler divergence (*D*_*KL*_) and is given as:

$$D_{JS}\left(P_{\text{emp}}|P_{\text{model}}\right)=\frac{1}{2}D_{KL}\left(P_{\text{emp}}|\left(P_{\text{model}}+P_{\text{emp}}\right)/2\right)+\frac{1}{2}D_{KL}\left(P_{\text{model}}|\left(P_{\text{model}}+P_{\text{emp}}\right)/2\right).$$

*MEM decoding*. We classified the cortical state from the spiking ensemble activity using a jackknife cross-validation approach. We used a total of *Q* = 10 random choices of ensembles of *N* signals. For each cortical state, 70% of the data was used to learn the parameters of a MEM (train-sets). The remaining data (test-sets) was associated to a cortical state by choosing the model with maximal goodness-of-fit (1/*D*_*JS*_) between the probability distribution of the test data and the learned model. The percentage of correct classifications was computed across all neuronal ensembles. The entire procedure was repeated 30 times with randomly selected train-sets to get confidence intervals of the classification performance. To assess statistical significance of the classification performance we calculated the probability of getting *k* correct classifications (hits) by chance, which is given by: $\text{Pr}\left(k\right)=C_{n}^{k}p^{k}{\left(1-p\right)}^{n-k}$, where *p* is the probability of getting a hit by chance (*p* = 1/*N*_*s*_, where *N*_*s*_ is the number of cortical states) and *n* is the number of tests (*n = QN*_*s*_). Significant decoding is reached when the decoding performance exceeds the 95% percentile of Pr(*k*).

#### Parameter re-scaling, heat capacity, and criticality

In the framework of statistical mechanics, one can characterize the physical state of the ensemble activity through the heat capacity. The heat capacity *C* is given by *C* = var[*E*]/T^2^ and it is calculated by introducing a “temperature” parameter T that acts as a scaling factor for all model parameters as **Ω** → **Ω**/T. In our study, we estimated the variance of the energies (*E*) from a large number of Monte Carlo simulations (5.10^5^ steps) for different T. A useful relation is the one linking the entropy and the heat capacity: the entropy *S* is the integral of the function *C*(T)/T from T = 0 to T = 1 [59]. As shown above (Fig 7D and 7E), a maximum of the heat capacity close to T = 1 suggests that the system is likely to be close to a critical state. Hence, *C* can be used as an additional diagnosis tool to assess criticality.

#### Neuronal network model

To test the effect of sub-sampling on the assessment of criticality, we modeled a network of 2500 integrate and fire neurons (70% excitatory, 30% inhibitory) which were arranged on a two-dimensional grid (50x50). Every neuron was assigned a spatial position (x,y) and connected to neighboring with connection probability (p) which decayed with distance according to Gaussian distribution: $p=\text{exp}\left(-\frac{{\left(x_{i}-x_{j}\right)}^{2}+{\left(y_{i}-y_{j}\right)}^{2}}{2\tau_{w}{}^{2}}\right)$, with spatial decay constant *τ*_*w*_ = 100 micrometers and a distance to the nearest neighbor of 25 microns [53,86].

The membrane potential *v*_*m*_ of each neuron evolves according to the following equation:

$$\frac{dv_{m}}{dt}=\frac{v_{rest}-v_{m}}{\tau_{m}}+\frac{g_{exc}\left(v_{exc}-v_{m}\right)+g_{inh}\left(v_{inh}-v_{m}\right)}{C},$$

where *v*_*rest*_ is the resting membrane potential with *v*_*exc*_ and *v*_*inh*_ representing the excitatory and inhibitory reversal potentials. The parameters *g*_*exc*_ and *g*_*inh*_ reflect the excitatory and inhibitory conductances, respectively and decay according to $\frac{dg_{exc}}{dt}=-g_{exc}\frac{1}{\tau_{exc}}$ and $\frac{dg_{inh}}{dt}=-g_{inh}\frac{1}{\tau_{inh}}$ with time constants *τ*_*exc*_ and *τ*_*inh*_ (see Table 3 for detailed values of neuronal parameters used in the simulation). The values of excitation and inhibition were chosen such that synchronized network activity emerged with power law statistics. A spike was emitted when the membrane potential reached a fixed threshold *v*_*th*_ of –50 mV and was reset to the resting membrane potential (-60 mV). Each neuronal discharge was followed by an absolute refractory period of 1ms. After a delay of 1ms, a spike triggered excitatory or inhibitory postsynaptic potentials in the receiving neurons, which decayed according to an exponential function.

**Table 3:** Neuronal parameters used for the spiking neuronal network model.

Neuronal parameter	Value	Description
**C**	200 pF	Membrane capacitance
**τ_m_**	20 ms	Membrane time constant
**v_rest_**	-60 mV	Resting membrane potential
**v_th_**	-50 mV	Firing threshold
**τ_exc_**	5 ms	Time constant excitatory synapses
**τ_inh_**	10ms	Time constant inhibitory synapses
**g_exc_**	0.7 nS	Excitatory peak conductance
**g_inh_**	50 nS	Inhibitory peak conductance
**v_exc_**	0 mV	Reversal potential of excitatory synapses
**v_inh_**	-70 mV	Reversal potential of inhibitory synapses

Each neuron received a constant external excitatory Poisson input *I*_*ext*_, adding the value $\frac{g_{exc}\left(v_{exc}-v_{m}\right)}{C}$ to the membrane potential for each Poisson spike. The rate of *I*_*ext*_ controls the state of the network: for low input (*I*_*ext*_ = 6,000 Hz) the network displayed patterned synchronized activity, for high input (*I*_*ext*_ = 9,000 Hz) the network was desynchronized. For each value of the external input, we performed two long simulations of 10 minutes. Thereafter, neurons were randomly removed from each simulation to test the consequences of sub-sampling for various network dynamic statistics. Each simulation was sub-sampled such that the population firing rate was reduced to 450Hz, 150Hz and 50Hz. For each condition we sub-sampled the network 50 times and subsequently average statistics were calculated. Standard deviations between the individual sub-sampling trials were exceedingly small, such that they were not plotted in Fig 6D, 6F and 6H. All simulations were performed using the Brian2 simulation environment [87].

#### Statistical analysis

Statistical differences between different cortical states were assessed using one-way repeated measures (rm) ANOVA followed by multiple comparisons using Bonferroni tests. The threshold for statistical significance was set to p-values<0.05. Where the ANOVA's sphericity assumption was not met (using the Mauchly test), p-values and degrees of freedom were corrected using the Huynh-Feldt estimates of sphericity (*ε*). The confidence intervals for the slope of least-squares linear fit was calculated as: $t_{n-2}\left(i\right)\sqrt{S^{2}/S_{xx}}$, where *n* is the number of data points, *S*^2^ is the unbiased estimator of the variance of the estimation errors, *S*_*xx*_ is the sum of squared deviations of observed predictors from their mean, t_*n*–2_(*i*) is the *i-*th percentile for the Student *t* distribution with (*n–* 2) degrees of freedom (we used 95% and 99%).

## Supporting information

S1 Fig Loglikelihood ratios for power law and lognormal fits to size distributions of different cortical states across all cat and monkey data and for different thresholds of spike cluster definition. Negative values indicate a better lognormal fit. State differences were assessed using a one-way rm-ANOVA test (threshold 1: cat: F_4,12_ = 4.03, p = 0.1, *ε* = 0.38; monkey: F_3,9_ = 24.57, p = 0.002, *ε* = 0.61; threshold 2: cat: F_4,12_ = 5.6, p = 0.03, *ε* = 0.6; monkey: F_3,9_ = 25.31, p = 0.0005, *ε* = 0.78; threshold 3: cat: F_4,12_ = 9.48, p = 0.005, *ε* = 1; monkey: F_3,9_ = 20.89, p = 0.001, *ε* = 0.73; threshold 4: cat: F_4,12_ = 3.68, p = 0.029, *ε* = 1; threshold 5: cat: F_4,12_ = 1.9, p = 0.12, *ε* = 1).
(TIF)

S2 Fig Loglikelihood ratios for power law and lognormal fits to liftime distributions of different cortical states across all cat and monkey data and for different thresholds of spike cluster definition. Negative values indicate a better lognormal fit. State differences were assessed using a one-way rm-ANOVA test (threshold 1: cat: F_4,12_ = 7.45, p = 0.02, *ε* = 0.6; monkey: F_3,9_ = 25.6, p = 0.002, *ε* = 0.58; threshold 2: cat: F_4,12_ = 5.21, p = 0.05, *ε* = 0.46; monkey: F_3,9_ = 27.02, p = 0.0006, *ε* = 0.74; threshold 3: cat: F_4,12_ = 12.2, p = 0.00003, *ε* = 1; monkey: F_3,9_ = 22.34, p = 0.002, *ε* = 0.63; threshold 4: cat: F_4,12_ = 2.03, p = 0.2, *ε* = 0.58; threshold 5: cat: F_4,12_ = 1.26, p = 0.34, *ε* = 0.98).
(TIF)

S3 Fig Avalanche shape collapse. **(A–B)** Averaged temporal profile of avalanche of lifetime Δ*t*, i.e., <*S*(*t*,Δ*t*)>, in the Desyn I cortical state (A) and the SynSlow cortical state (B) for an example cat dataset. **(C–D)** Scaled avalanche profiles as a function of the scaled time *t*/Δ*t*, in the Desyn I cortical state (C) and the SynSlow cortical state (D). Red line: averaged scaled avalanche profile; σνz: best scaling parameter. **(E)** Collapse index (CI) for each cortical state, averaged over all cat datasets (F_4,12_ = 1.53, p = 0.254; *ε* = 1). Error bars indicate SEM. **(F)** CI calculated by grouping the avalanches of the desynchronized cortical states (Desyn I/II) and, separately, those of the synchronized states (SynSlow I/II) (p = 0.027, paired t-test). Error bars indicate SEM. **(G–H)** Averaged temporal profile of avalanche of lifetime Δ*t* of avalanches display by the spiking model in the desynchronized states (G) and in the synchronized state (H). **(I–J)** Scaled avalanche profiles for the spiking model in the desynchronized state (I) and the synchronized state (J).
(TIF)

S4 Fig Separation of different states within the synchronized state of the model. **(A-B)** Mean Fano factor (FF) for different states in cat and monkey recordings (bin-size = 100ms). Error bars indicate SEM. **(C)** Distribution of FFs computed for each one second segment of the modeled synchronized state (bin-size = 50ms). **(D)** Mean firing rate of model neurons for different parts of the FF distribution. **(E)** Cluster size distributions of different levels of synchronization in the SI state defined by the FF distribution.
(TIF)

S1 Dataset Spiking data from four cat and four monkey datasets. The data include the spike timings, electrode index and state information. Details can be found in the README file.
(ZIP)

S1 Text Shape collapse and state analysis of the synchronized model state. (DOCX)
