# Supplementary figures and images for: Spontaneous cortical activity is transiently poised close to criticality
Source: PLoS Comput Biol. 2017 May 24;13(5):e1005543. doi: 10.1371/journal.pcbi.1005543 (PMC5464673; doi:10.1371/journal.pcbi.1005543)

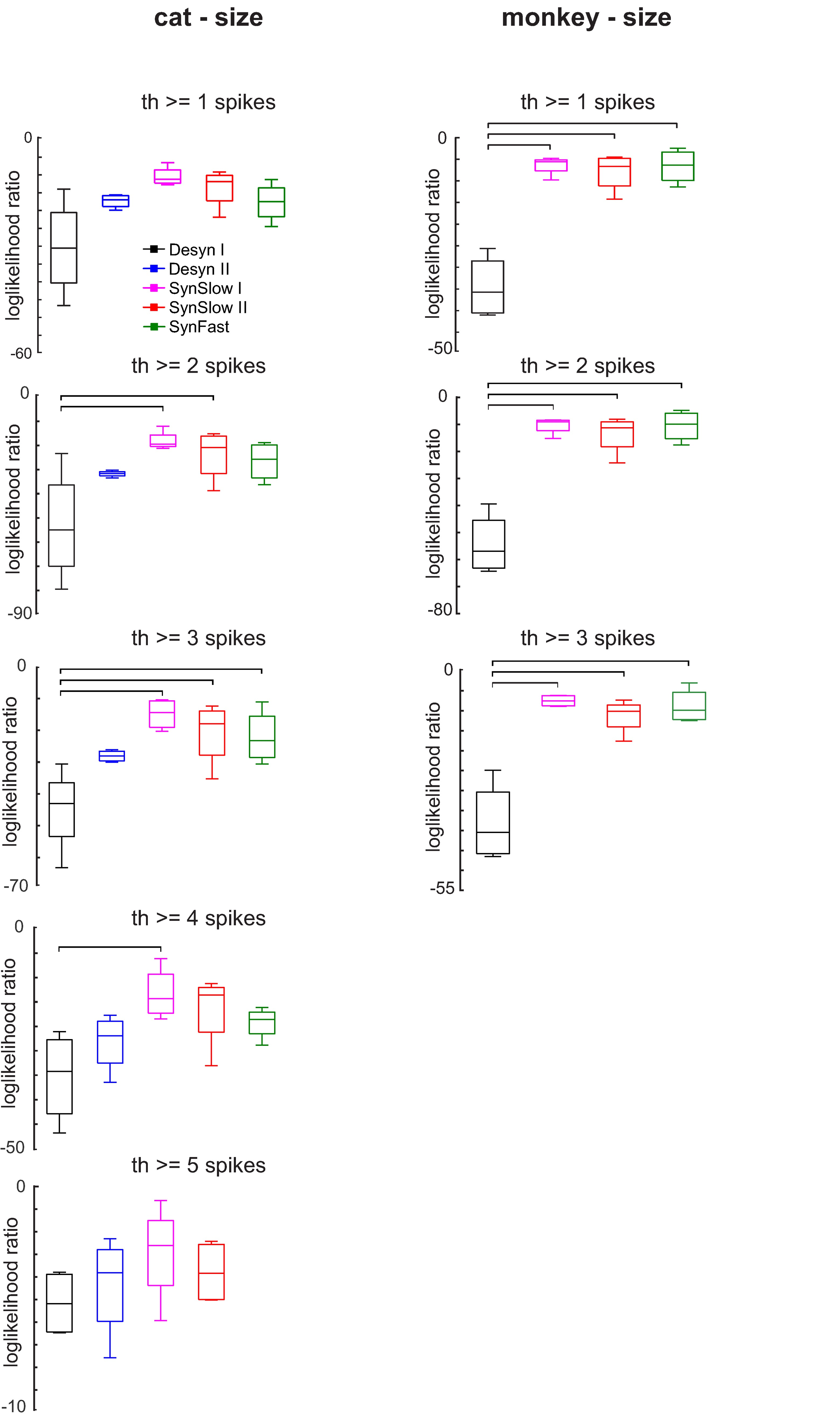

Supplement: S1 Fig — Negative values indicate a better lognormal fit. State differences were assessed using a one-way rm-ANOVA test (threshold 1: cat: F4,12 = 4.03, p = 0.1, ε = 0.38; monkey: F3,9 = 24.57, p = 0.002, ε = 0.61; threshold 2: cat: F4,12 = 5.6, p = 0.03, ε = 0.6; monkey: F3,9 = 25.31, p = 0.0005, ε = 0.78; threshold 3: cat: F4,12 = 9.48, p = 0.005, ε = 1; monkey: F3,9 = 20.89, p = 0.001, ε = 0.73; threshold 4: cat: F4,12 = 3.68, p = 0.029, ε = 1; threshold 5: cat: F4,12 = 1.9, p = 0.12, ε = 1). (TIF) [file pcbi.1005543.s001.tif]

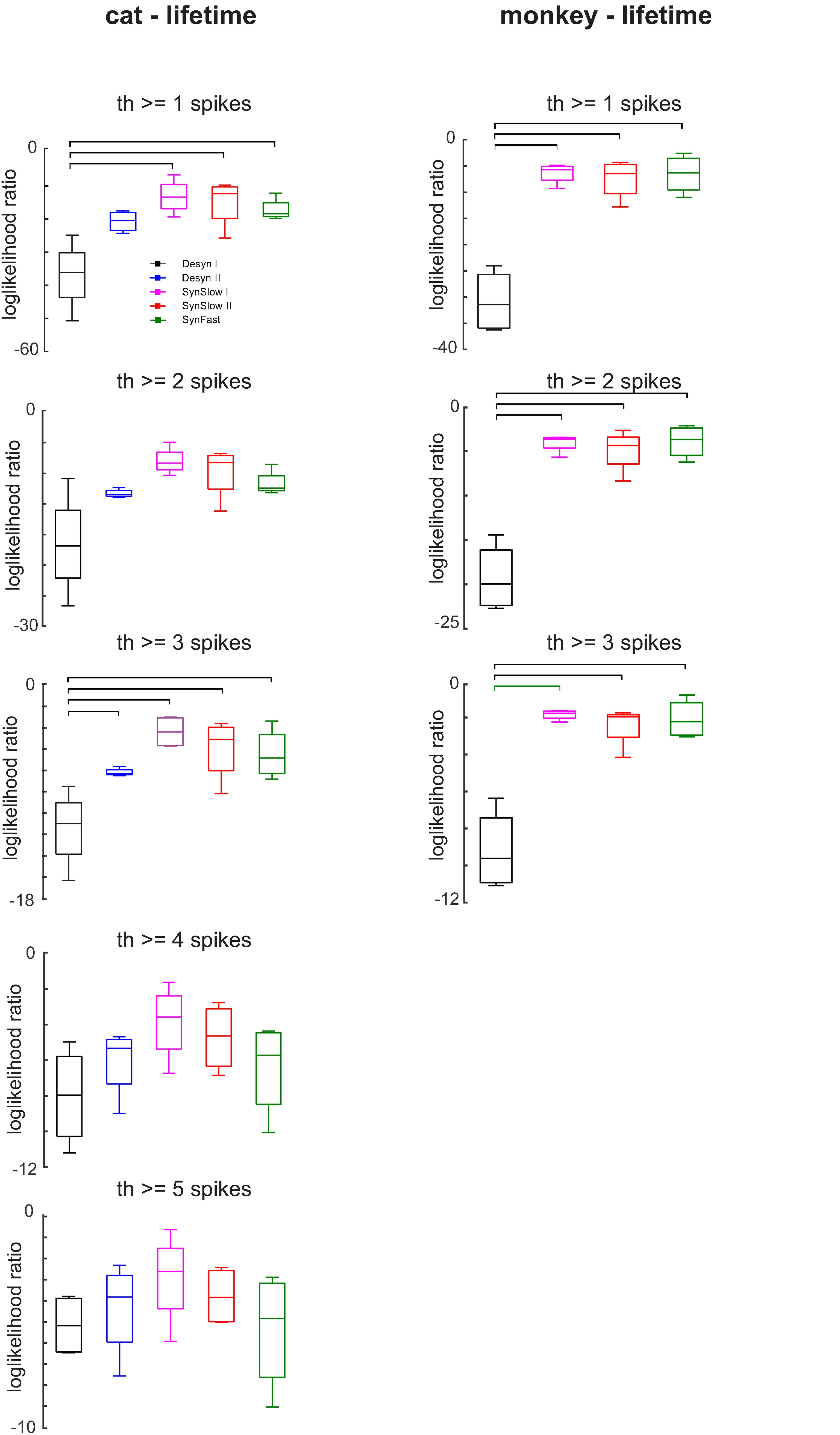

Supplement: S2 Fig — Negative values indicate a better lognormal fit. State differences were assessed using a one-way rm-ANOVA test (threshold 1: cat: F4,12 = 7.45, p = 0.02, ε = 0.6; monkey: F3,9 = 25.6, p = 0.002, ε = 0.58; threshold 2: cat: F4,12 = 5.21, p = 0.05, ε = 0.46; monkey: F3,9 = 27.02, p = 0.0006, ε = 0.74; threshold 3: cat: F4,12 = 12.2, p = 0.00003, ε = 1; monkey: F3,9 = 22.34, p = 0.002, ε = 0.63; threshold 4: cat: F4,12 = 2.03, p = 0.2, ε = 0.58; threshold 5: cat: F4,12 = 1.26, p = 0.34, ε = 0.98). (TIF) [file pcbi.1005543.s002.tif]

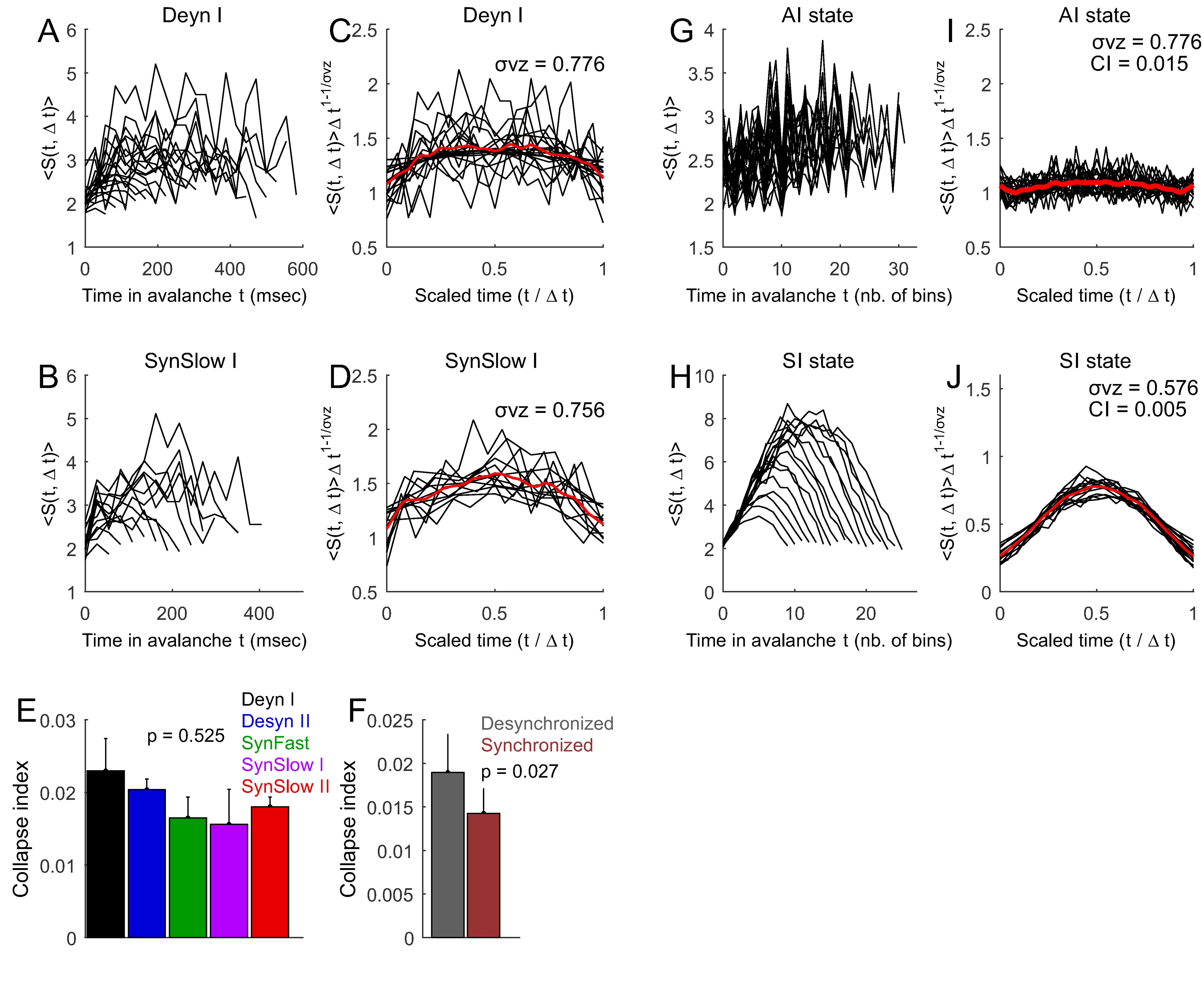

Supplement: S3 Fig — (A–B) Averaged temporal profile of avalanche of lifetime Δt, i.e., <S(t,Δt)>, in the Desyn I cortical state (A) and the SynSlow cortical state (B) for an example cat dataset. (C–D) Scaled avalanche profiles as a function of the scaled time t/Δt, in the Desyn I cortical state (C) and the SynSlow cortical state (D). Red line: averaged scaled avalanche profile; σνz: best scaling parameter. (E) Collapse index (CI) for each cortical state, averaged over all cat datasets (F4,12 = 1.53, p = 0.254; ε = 1). Error bars indicate SEM. (F) CI calculated by grouping the avalanches of the desynchronized cortical states (Desyn I/II) and, separately, those of the synchronized states (SynSlow I/II) (p = 0.027, paired t-test). Error bars indicate SEM. (G–H) Averaged temporal profile of avalanche of lifetime Δt of avalanches display by the spiking model in the desynchronized states (G) and in the synchronized state (H). (I–J) Scaled avalanche profiles for the spiking model in the desynchronized state (I) and the synchronized state (J). (TIF) [file pcbi.1005543.s003.tif]

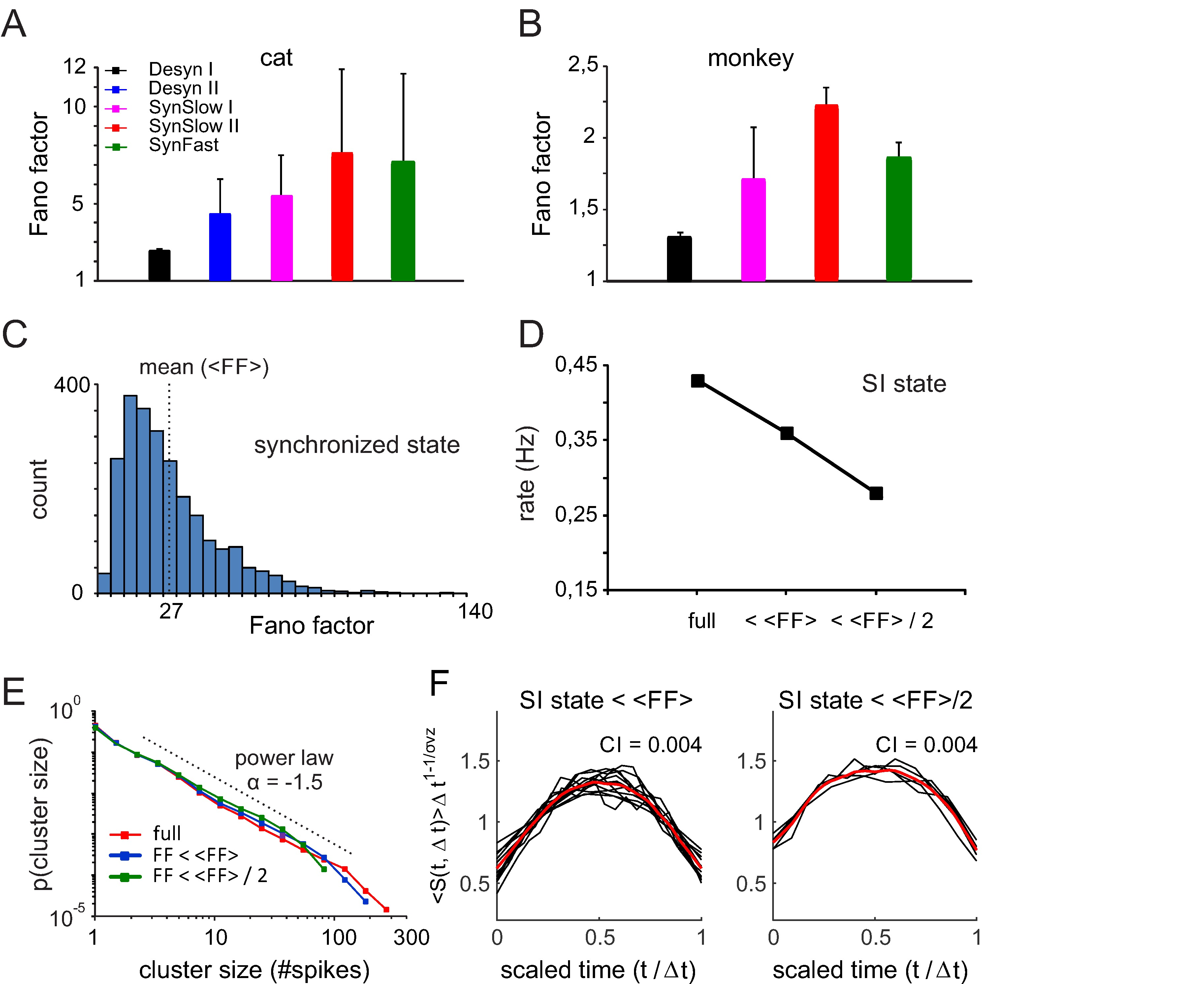

Supplement: S4 Fig — (A-B) Mean Fano factor (FF) for different states in cat and monkey recordings (bin-size = 100ms). Error bars indicate SEM. (C) Distribution of FFs computed for each one second segment of the modeled synchronized state (bin-size = 50ms). (D) Mean firing rate of model neurons for different parts of the FF distribution. (E) Cluster size distributions of different levels of synchronization in the SI state defined by the FF distribution. (TIF) [file pcbi.1005543.s004.tif]
