# Supplementary material for: Spontaneous cortical activity is transiently poised close to criticality
Source: PLoS Comput Biol. 2017 May 24;13(5):e1005543. doi: 10.1371/journal.pcbi.1005543 (PMC5464673; doi:10.1371/journal.pcbi.1005543)
Supplement: S1 Text — (DOCX) [file pcbi.1005543.s006.docx]

**S1 Text**

**Avalanche shape collapse**

We investigated whether the observed neuronal avalanches produced self-similar dynamics as predicted by criticality theory. The theory predicts that the averaged time-course of avalanches of lifetime Δt, i.e., $\left\langle S(t,\Delta t) \right\rangle$, where $S\left( t,\Delta t \right)$ is the size of the avalanche at time *t* ($1\leq t\leq\Delta t$), does not depend on Δ*t* in the scaled axes given by the normalize time $t/\Delta t$ and the scaled avalanche profile $\left\langle S(t,\Delta t) \right\rangle{\Delta t}^{1-1/{\sigma\nu z}}$. In other words $\left\langle S(t,\Delta t) \right\rangle{\Delta t}^{1-1/{\sigma\nu z}}=F(t/{\Delta t})$, where the function *F* does not depend on the temporal scale and the exponent $\sigma\nu z$ satisfies the relation: $\left( \tau-1 \right)/\left( \alpha-1 \right)=\sigma\nu z$, where $\alpha$ is the power law exponent of the distribution of avalanche lifetimes and $\tau$ is the power exponent of the distribution of avalanche sizes. This invariance across scales is known as “shape collapse”.. We used the method of Marshall et al. [1] to automatically find the scaling parameter $a=1-1/{\sigma\nu z}$ that produces the best possible collapse, i.e., the parameter $a$ that minimizes the variance across the avalanche profiles in the normalized time ($t/\Delta t$). The minimized variance was used as a collapse index (CI) that tends to 0 when scaled avalanche profiles collapse into a single curve. Since this analysis requires a good estimation of $\left\langle S(t,\Delta t) \right\rangle$, we only analyzed the cat datasets, for which more data was available, and we restricted the analysis to temporal scales (Δ*t*) for which at least 10 avalanches were observed.

In our data, the avalanche time-courses (S3A-B Figs) did not perfectly collapse for none of the states (S3C-D Figs), probably due to the limited number of avalanches in each cortical state. However, the empirical avalanche profiles tended to collapse more for the synchronized states than for the desynchronized states. Differences in CI were statistically different when grouping the data of Desyn I/II and SynSlow I/II (p = 0.027, paired t-test, S3F Fig), otherwise, CI was reduced from the synchronized to desynchronized states but differences were not significant (p = 0.254, one-way rm-ANOVA, S3E Fig). Further studies, with larger amount of data, are needed to test self-similar dynamics in different cortical states.

We also performed the analysis on the simulated data using the spiking model. As shown in S3G-J Figs, data generated by the model in the desynchronized state do not collapse, while data generated in the synchronized state does. This result is in line with our observation that empirical data from desynchronized states lead to a higher variance (i.e., CI) than empirical data from synchronized states.

**State analysis of the synchronized model state**

We tested whether differences found in various criticality metrics across cortical states in our data are a byproduct of artificially separating critical dynamics into states with varying levels of population synchronization. Similar to previous studies [2], we delineated states of different synchronization levels in the model SI state based on population spike count variability and quantified synchronization by the Fano factor [3], which is defined as: FF = var(spike count) / mean(spike count), with the spike count reflecting the sum of spikes across the entire neuronal population within a given bin of the binned datasets. The bin-size was chosen as 100ms for the experimental data and 50ms for the model. When analyzing the Fano factors of the already separated states in both cat and monkey datasets, the FFs of spiking activity averaged across the 1 second data segments were close to one for desynchronized activity as expected from an independent Poisson process, while synchronized states were associated with FFs >>1 ( S4A-B Figs). These results demonstrate that differences in FF describe different states in our data. We then split the synchronized model state into bins (250ms duration) similar to the experimental data and calculated the FF for each bin. The FF distribution of the critical SI state indeed showed a large range of different synchronization levels (S4C Fig). To test whether more desynchronized activity in the SI state is accompanied by a loss of power law as seen in the experimental data, we successively removed synchronized bins such that only bins with FF less than the mean or mean/2 of the FF distribution were analyzed. While the mean spike rate of the remaining data decreased (S4D Fig), the power law was preserved even in the absence of strongly synchronized states (S4E Fig). The manipulation only moved the power law cutoff to smaller avalanches. In addition, results of the collapse analysis for the more desynchronized model SI activity were indistinguishable from the fully sampled case (S3J and S4F Figs).

1. Marshall N, Timme NM, Bennett N, Ripp M, Lautzenhiser E, Beggs JM. Analysis of Power Laws, Shape Collapses, and Neural Complexity: New Techniques and MATLAB Support via the NCC Toolbox. Front Physiol. 2016;7: 250. doi:10.3389/fphys.2016.00250

2. Renart A, De la Rocha J, Bartho P, Hollender L, Parga N, Reyes A, et al. The asynchronous state in cortical circuits. Science (80- ). American Association for the Advancement of Science; 2010;327: 587. Available: http://www.sciencemag.org/content/327/5965/587.short

3. Kumar A, Rotter S, Aertsen A. Conditions for propagating synchronous spiking and asynchronous firing rates in a cortical network model. J Neurosci. 2008;28: 5268–80. doi:10.1523/JNEUROSCI.2542-07.2008
